# Microglial–oligodendrocyte interactions in myelination and neurological function recovery after traumatic brain injury

**DOI:** 10.1186/s12974-022-02608-6

**Published:** 2022-10-05

**Authors:** Shanshan Song, Md Nabiul Hasan, Lauren Yu, Satya S. Paruchuri, John P. Bielanin, Shamseldin Metwally, Helena C. M. Oft, Sydney G. Fischer, Victoria M. Fiesler, Tanusree Sen, Rajaneesh K. Gupta, Lesley M. Foley, T. Kevin Hitchens, C. Edward Dixon, Franca Cambi, Nilkantha Sen, Dandan Sun

**Affiliations:** 1 Department of Neurology, University of Pittsburgh, 3501 Fifth Avenue, Pittsburgh, PA 15213 USA; 2 Pittsburgh Institute for Neurodegenerative Disorders, University of Pittsburgh, Pittsburgh, PA 15213 USA; 3 Veterans Affairs Pittsburgh Health Care System, Pittsburgh, PA 15213 USA; 4 Department of Neurological Surgery, University of Pittsburgh, Pittsburgh, PA 15213 USA; 5 Animal Imaging Center, University of Pittsburgh, Pittsburgh, PA 15213 USA; 6 Department of Neurobiology, University of Pittsburgh, Pittsburgh, PA 15213 USA

**Keywords:** White matter damage, Inflammation, Oligodendrocytes, Microglia, Na^+^/H^+^ exchanger, Traumatic brain injury

## Abstract

Differential microglial inflammatory responses play a role in regulation of differentiation and maturation of oligodendrocytes (OLs) in brain white matter. How microglia–OL crosstalk is altered by traumatic brain injury (TBI) and its impact on axonal myelination and neurological function impairment remain poorly understood. In this study, we investigated roles of a Na^+^/H^+^ exchanger (NHE1), an essential microglial pH regulatory protein, in microglial proinflammatory activation and OL survival and differentiation in a murine TBI model induced by controlled cortical impact. Similar TBI-induced contusion volumes were detected in the *Cx3cr1-Cre*^*ERT2*^ control (Ctrl) mice and selective microglial *Nhe1* knockout (*Cx3cr1-Cre*^*ERT2*^*;Nhe1*^*flox/flox*^, *Nhe1* cKO) mice. Compared to the Ctrl mice, the *Nhe1* cKO mice displayed increased resistance to initial TBI-induced white matter damage and accelerated chronic phase of OL regeneration at 30 days post-TBI. The cKO brains presented increased anti-inflammatory phenotypes of microglia and infiltrated myeloid cells, with reduced proinflammatory transcriptome profiles. Moreover, the cKO mice exhibited accelerated post-TBI sensorimotor and cognitive functional recovery than the Ctrl mice. These phenotypic outcomes in cKO mice were recapitulated in C57BL6J wild-type TBI mice receiving treatment of a potent NHE1 inhibitor HOE642 for 1–7 days post-TBI. Taken together, these findings collectively demonstrated that blocking NHE1 protein stimulates restorative microglial activation in oligodendrogenesis and neuroprotection, which contributes to accelerated brain repair and neurological function recovery after TBI.

**Supplementary Information:**

The online version contains supplementary material available at 10.1186/s12974-022-02608-6.

## Background

Traumatic brain injury (TBI) causes acute apoptotic death of mature oligodendrocytes (OLs) in the white matter tracts [1], which contributes to prolonged cognitive [2, 3], perceptual [4], and sensorimotor deficits [5]. Despite recruitment and accumulation of oligodendrocyte progenitor cells (OPCs) to the injury site from day 2 to 3 months post-injury [1] in white matter repair [6], remyelination mediated by this process is inefficient and often fails due to inadequate OPC differentiation [7], causing sustained axonal demyelination in TBI patients up to 5 years post-TBI [8]. To date, no effective treatments are currently available to reduce TBI-induced OL death and/or to stimulate OPC differentiation for white matter repair. A better understanding of the underlying mechanisms of TBI-mediated white matter injury and the identification of new therapeutic targets to stimulate remyelination are warranted. Reducing OL death and white matter damages while promoting differentiation of OPCs into mature myelinating OLs emerge as a central strategy for improving white matter repair and neurological function recovery after TBI.

Microglia play an important role in supporting normal myelin genesis during development and adulthood, as depletion of microglia either in early postnatal stage or in adulthood significantly reduced OPC/OL numbers and inhibited myelin formation in the corpus callosum (CC) and white matter regions of cerebellum [9]. Restorative phenotype activation of microglia increases secretion of restorative cytokines/growth factors (TGF-β, IL-10, BDNF, GDNF) and clearing of tissue debris through phagocytosis, which stimulates OL genesis and differentiation for remyelination and brain functional recovery [10, 11]. However, activation of microglial inflammation causes secondary damage, which can directly trigger OL apoptosis and hinder OPC maturation, prolonging post-TBI demyelination [10, 11]. Thus, maintaining a balanced microglial activation phenotype homeostasis is important for OL survival, differentiation, and myelination [12].

We recently discovered that activation of microglial NHE1 protein, which mediates H^+^ efflux in exchange of Na^+^ influx, maintains the optimal alkaline intracellular pH (pH_i_) for sustained activation of NADPH oxidase (NOX2) and cytokine release in proinflammatory microglia [13, 14]. However, whether microglial NHE1 protein plays a role in regulating microglia-mediated inflammation and microglia–OL interactions after TBI has not been investigated. In this study, we found that either selective deletion of microglial *Nhe1* in the *Cx3cr1-Cre*^*ERT2*^*;Nhe1*^*flox/flox*^ (*Nhe1* cKO) mice or pharmacological inhibition of NHE1 protein with its potent inhibitor HOE642 accelerated post-TBI functional recovery, in comparison to the *Cx3cr1-Cre*^*ERT2*^ control (Ctrl) or vehicle-treated wild-type (WT) mice. The cKO mice not only exhibited increased tolerance to acute post-TBI neural degeneration and white matter damage, but also showed accelerated regeneration of the OLs and white matter remyelination at chronic phase post-TBI. Flow cytometry and bulk RNAseq of microglia/macrophages revealed reduced inflammatory responses and increased restorative phenotypes in the cKO brains. Taken together, our findings strongly suggest that targeting NHE1 protein emerges as a novel therapeutic strategy for modulating restorative microglial activation in enhancing neuroprotection and oligodendrogenesis in post-TBI tissue repair and neurological function recovery.

## Material and methods

### Animals

All animal studies were approved by the University of Pittsburgh Institutional Animal Care and Use Committee, which adhere to the National Institutes of Health Guide for the Care and Use of Laboratory Animals and reported in accordance with the Animal Research: Reporting In Vivo Experiments (ARRIVE) guidelines [15]. Tamoxifen-injected *Cx3cr1-Cre*^*ER*+/−^; *Nhe1*^*flox/flox*^ mice and *Cx3cr1-Cre*^*ER*+/−^ mice were used as cKO and Ctrl groups, as we recently described [16]. For the inhibitor study, a potent NHE1 inhibitor HOE642 (Sigma-Aldrich, USA) was administered twice per day by i.p. injections from day 1–7 post-TBI in C57BL/6 male mice, as described in Additional file 1.

### Controlled cortical impact (CCI)-induced TBI procedures

Mice were anesthetized and subjected to CCI as described [17]. Briefly, mice were placed in a stereotaxic frame (Leica, Germany) and impacted at 4.5 m/s with 20 ms dwell time and 1.2 mm depression, mimicking a moderate TBI. Sham animals underwent the same procedures without the impact, as described in Additional file 1.

### Behavioral function tests

Neurological functional deficits in mice were screened in a blinded manner with adhesive contact/removal test, foot fault test, and y-maze test, all considered reliable for identifying and quantifying sensorimotor and cognitive deficits in mouse models of TBI [18–20]. Please see Additional file 1 for detailed information.

### MRI and DTI of ex vivo brains

At 30 days post-TBI, the same cohort of mice from the behavioral assessments were transcardially perfused with 4% paraformaldehyde (PFA), and ex vivo brains were collected for MRI and diffusion tensor imaging (DTI), as described in Additional file 1. Corpus callosum (CC) and external capsules (EC) were drawn in contralateral (CL) and ipsilateral (IL) hemispheres and values of fractional anisotropy (FA) were calculated, as described before [21].

### Flow cytometry profiling of microglia/macrophages

Single cell suspensions were obtained from CL and IL hemispheric tissues using a neural tissue dissociation kit with the gentleMACS Octo Dissociator (Miltenyi Biotec Inc., Germany), as described before [16, 21]. Cells were stained with antibodies listed in Additional file 1. Data were acquired using an LSRII flow cytometer (BD Biosciences, USA) and analyzed with Flow Jo (Tree Star Inc, USA) software.

### Magnetic-activated cell sorting (MACS) isolation of microglia/macrophages

CD11b^+^ microglia/macrophages were isolated from CL and IL single cell suspensions by MACS using the CD11b MicroBeads (Miltenyi Biotech, USA). Detailed information is described in Additional file 1.

### Bulk RNA sequencing and bioinformatics analysis

Bulk RNA sequencing was performed in MACS-isolated CD11b^+^ microglia/macrophages and paired-end sequenced on Illumina NovaSeq platform using a Smart Seq v4 library preparation kit, as in our recent report [22]. Bioinformatic analysis was performed using Partek Flow 8.0 software (Partek, USA), as described in Additional file 1. The sequencing data have been deposited in the Gene Expression Omnibus (GEO) database with experiment series accession number GSE199869.

### Quantitative real-time PCR

RNA was isolated from the MACS-isolated CD11b^+^ microglia/macrophages using a Direct-zol RNA MicroPrep Kit (Zymo Research, USA), following the manufacturer’s instruction. qPCR was performed on a CFX96 Real-Time PCR Detection System (Bio-rad, USA), as described in Additional file 1. Data were analyzed using the ΔΔCt method [23] with triplicate reactions for each gene evaluated. Primer sequences are listed in Additional file 1: Table S1.

### Immunofluorescent staining

Mouse brains were fixed with transcardial perfusion with 4% PFA, and cryosectioned at 25 μm thickness for immunofluorescent staining, as described in Additional file 1. Identical acquisition parameters were used and fluorescent images were obtained using a Nikon A1R confocal microscope (Nikon, Japan) before analyzing with ImageJ (NIH, USA).

### Statistical analysis

Unbiased study design and analyses were used in all the experiments. Blinding of investigators to experimental groups were maintained until data were fully analyzed whenever possible. Power analysis were performed based on the mean and variability of data from our laboratory. *N* = 8 mice/group for behavioral tests, *N* = 6 for immunostaining, flow cytometry, qPCR, and *N* = 4 for RNAseq and MRI/DTI were sufficient to give us 80% power to detect 20% changes with 0.05 two-sided significance. Data were expressed as mean ± SEM. Two-tailed Student’s *t*-test with 95% confidence was used when comparing two conditions. For more than two conditions, two-way ANOVA analysis was used. *p* value < 0.05 was considered statistically significant (Prism, GraphPad, USA).

## Results

### Microglial *Nhe1* cKO mice exhibited accelerated sensorimotor and cognitive function recovery after TBI

**Fig. 1:**
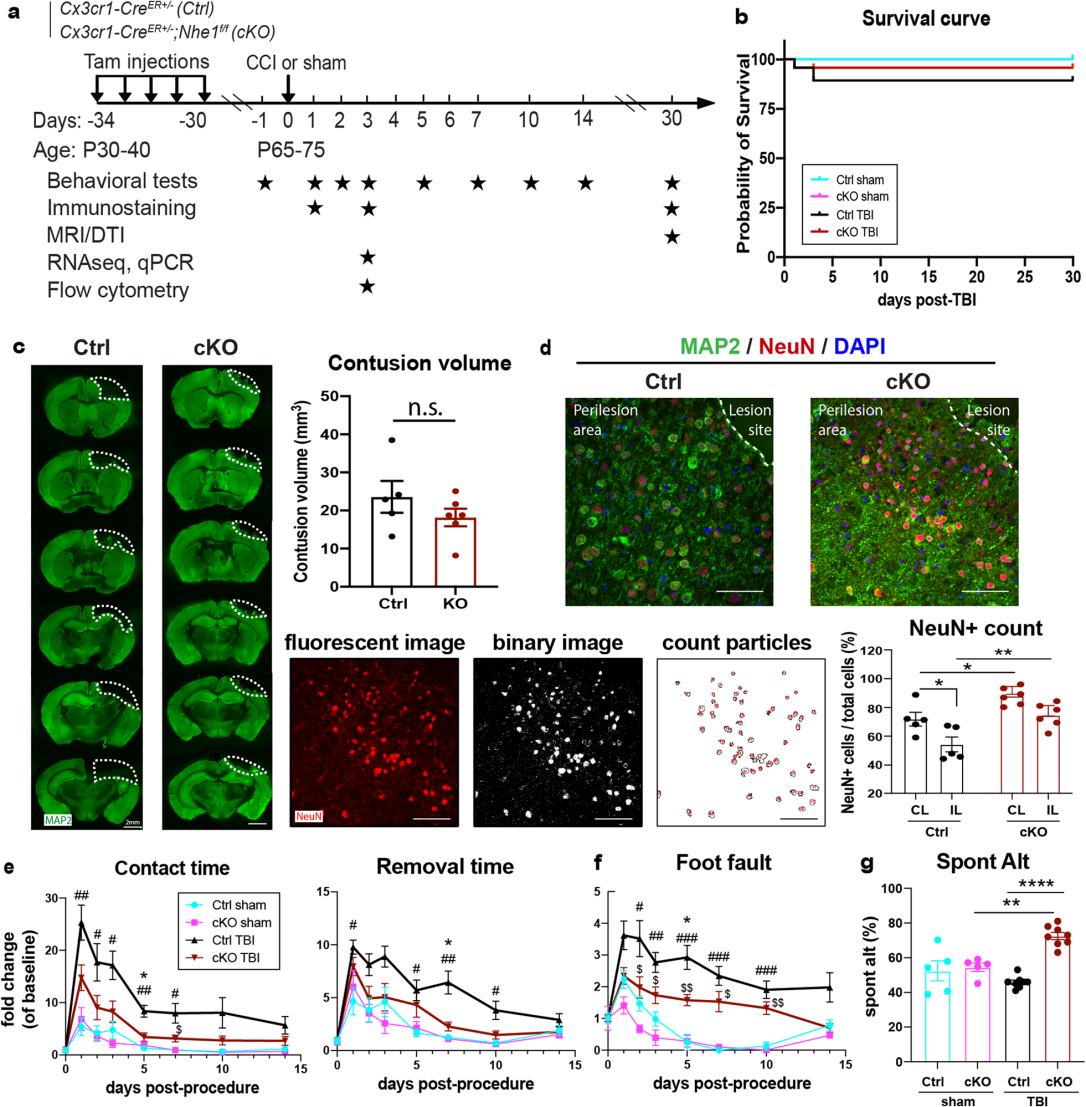
Effects of microglial *Nhe1* deletion on contusion volume and neurological function recovery in post-TBI mice. **a** Experimental protocol. *Cx3cr1-Cre*^*ER*+/−^ control (Ctrl) mice or *Cx3cr1-Cre*^*ER*+/−^*;Nhe1*^*f/f*^ (*Nhe1* cKO) mice at postnatal day 30–40 (P30-40) were given tamoxifen (Tam, 75 mg/kg body weight/day at a concentration of 20 mg/ml in corn oil, intraperitoneally) for five consecutive days. A 30-day post-injection waiting period was used for clearance of Tam and for replenishing of peripheral *Cx3cr1*^+^ monocytes prior to the induction of CCI or sham procedures. **b** Survival curve of Ctrl and cKO mice during 1–30 days post-sham or TBI. **c** Contusion volume of Ctrl or cKO brains at 3 days post-TBI assessed by neuronal marker MAP2 expression. **d** Representative images of neuronal marker MAP2 and NeuN expressions with unbiased automatic quantification of NeuN^+^ neurons in the contralateral (CL) and the ipsilateral (IL) peri-lesion cortex of Ctrl or cKO brains at 3 days post-TBI. N = 5 for Ctrl (3 males, 2 females), and N = 6 for cKO (4 males, 2 females). Scale bar = 50 µm**. e** Adhesive tape removal test in mice at 1–14 d post-sham or TBI. **f.** Foot fault test in the same cohort of mice as in e. **g.** Y-maze test in the same cohort of mice as in e. Data are mean ± SEM. N = 5 for sham groups, N = 8 for TBI groups (all males). * *p* < 0.05, ** *p* < 0.01, **** *p* < 0.0001, Ctrl TBI vs. cKO TBI. # p < 0.05, ## *p* < 0.01, ### *p* < 0.001 Ctrl TBI vs. Ctrl sham. $ p < 0.05, $$ p < 0.01, cKO TBI vs. cKO sham

We demonstrated in our recent report that our *Nhe1* cKO mouse line successfully deleted NHE1 protein expression exclusively in the IBA1^+^ microglia/macrophages, but remained unchanged in other cell types [21]. Survival rate and neurological behavior functions in Ctrl and *Nhe1* cKO mice were monitored during 1–30 days post-TBI (Fig. 1a). Neither Sham Ctrl or Sham cKO mice displayed any mortality, while Ctrl and cKO TBI mice exhibited < 10% mortality (Fig. 1b). Ctrl and cKO TBI mice showed similar contusion volume initially at 3 days post-TBI (Fig. 1c). However, the unbiased analysis of NeuN^+^ neuronal counts by automatic cell counting (using the “count particles” module in ImageJ) revealed significantly higher NeuN^+^ cell percentages in both CL and IL peri-lesion cortex of the cKO brains than the Ctrl brains (Fig. 1d). Moreover, compared to the Ctrl TBI mice, significantly increased NeuN intensity in the whole hippocampus area and NeuN^+^ cell counts in CA1, CA3 and dentate gyrus regions were detected in the cKO TBI mice (Additional file 1: Fig. S1). In assessing their correlation to neurological functional outcomes, Ctrl and cKO sham animals showed a brief elevation of sensorimotor deficits at 1 day post-sham, but quickly returned to baseline at 3 days post-procedure (Fig. 1e, f). In comparison, the Ctrl TBI mice exhibited significantly prolonged contact and removal time (~ 20-fold and 10-fold, respectively), as well as significantly more errors in the foot fault test (~ 4-fold) at 1–7 days post-TBI (Fig. 1e, f). However, compared to the Ctrl TBI mice, the *Nhe1* cKO mice showed significantly accelerated sensorimotor function recovery during 5–14 days post-TBI, and completely returned to their baseline levels by day 14 (Fig. 1e, f). In testing working memory using the Y-maze test at 30 days post-TBI, the cKO mice exhibited a significantly higher spontaneous alternation rate (~ 73%) than the Ctrl mice (~ 46%, *p* < 0.0001), indicating a stimulated working memory function in the cKO mice (Fig. 1g). However, the two groups displayed similar locomotor activities reflected by total arm entries (Additional file 1: Fig. S2). Taken together, these neurological function assessment tests demonstrate that the *Nhe1* cKO mice exhibited better neurological function (sensorimotor and cognitive) recovery after TBI, associated with preservation of cortical and hippocampal neurons.

### Microglial *Nhe1* cKO mice displayed improved white matter resistance against TBI-induced apoptosis and inhibition of oligodendrogenesis

**Fig. 2:**
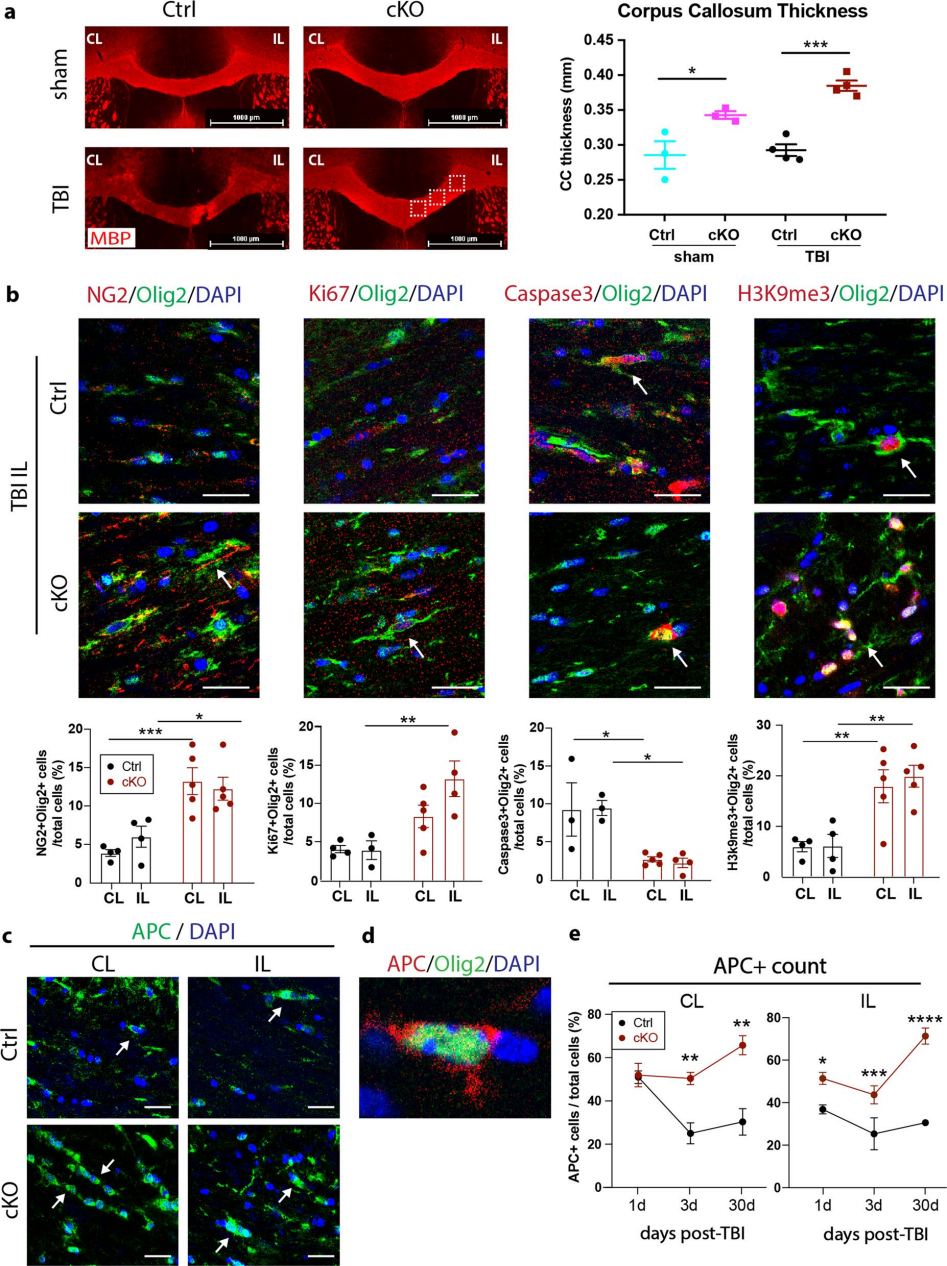
Microglial *Nhe1* cKO mice increased white matter tolerance to TBI with enhanced oligodendrogenesis and differentiation. **a.** Representative immunofluorescent images of MBP staining (at same bregma level) showing the cKO mice exhibited increased corpus callosum (CC) thickness at 1 day after sham or CCI procedures. *N* = 3 for Ctrl sham (1 male, 2 females) and cKO sham (1 male, 2 females); *N* = 4 for Ctrl TBI (2 males, 2 females) and cKO TBI (2 males, 2 females). Boxed areas indicate locations where zoom-in images (in b and c) were collected. **b.** Representative images and quantification of Olig2 colocalized with NG2, Ki67, Caspase-3, and H3K9me3 in CC of Ctrl or cKO brains at 3 days post-TBI. *N* = 4 for Ctrl (2 males, 2 females) and *N* = 5 for cKO (3 males and 2 females). Scale bar = 10 µm. **c.** Representative images of APC^+^ mature oligodendrocytes in the CC of Ctrl or cKO brains at 3 days post-TBI. Arrows: APC^+^ mature OLs. Scale bar = 10 µm. **d.** Colocalization of Olig2^+^ with the APC^+^ mature oligodendrocytes. **e.** Quantification of APC^+^ mature oligodendrocytes in the CC of Ctrl or cKO brains at 1, 3, and 30 days post-TBI. * *p* < 0.05, ** *p* < 0.01, *** *p* < 0.001, **** *p* < 0.0001

As white matter integrity is important for restoring neurological functions after TBI [2, 8], we tested whether the improved functional outcomes of the cKO mice were in part due to their increased tolerance to TBI-induced damage and/or boosted white matter repair. Myelin basic protein (MBP), a marker for white matter myelination, was used to assess corpus callosum (CC) tract integrity in the Ctrl and cKO brains after sham or TBI procedures. Interestingly, the cKO brains exhibited a thicker CC (midline, same bregma level, Additional file 1: Fig. S3) at 24 h after sham procedure (Fig. 2a, *p* < 0.05), indicating a possible role of microglial NHE1 protein in regulating white matter integrity homeostasis. At 1 day post-TBI, the cKO brains showed a significantly higher CC thickness than the Ctrl brains (Fig. 2a, *p* < 0.001). Further analysis of Olig2^+^ oligodendrocyte lineage cells at 3 days post-TBI revealed significantly elevated NG2^+^Olig2^+^ OPCs, Ki67^+^Olig2^+^ proliferative OLs, and reduced Caspase3^+^Olig2^+^ apoptotic OLs in both hemispheres of the cKO brains, compared to Ctrl brains (Fig. 2b, *p* < 0.05). Importantly, these cKO brains also exhibited increased expression of H3K9me3 in the Olig2^+^ OLs, a post-translational histone modification marker for OPC differentiation [24] (Fig. 2b, *p* < 0.01). Moreover, analysis of APC^+^ mature OLs counts showed that TBI did not affect OL survival in the CL hemispheres of either Ctrl or cKO brains, but induced an immediate decrease of the APC^+^ mature OLs in the IL hemisphere of the Ctrl mice at 1 d post-TBI (Fig. 2c–e, *p* < 0.01). In contrast, the cKO TBI brains were resistant to such a loss in the IL hemisphere (Fig. 2c–e). The Ctrl TBI mice continued to lose mature OLs in both CL and IL CC at 3 days post-TBI, while the TBI-induced reduction of mature OLs in the cKO CC was delayed (Fig. 2c–e). Interestingly, by 30 days post-TBI, the mature OLs in Ctrl mice failed to regenerate, while the cKO brains exhibited significantly elevated counts of mature OLs in both hemispheres of the CC tracks (Fig. 2e, *p* < 0.01). These findings strongly suggest that deletion of microglial NHE1 protein not only provided resistance to the white matter damage induced by TBI, but also promoted oligodendrogenesis by increasing their progenitor cell proliferation and differentiation into mature myelinating OLs.

### Selective deletion of microglial *Nhe1* increased microglial anti-inflammatory phenotype activation in TBI brains

**Fig. 3:**
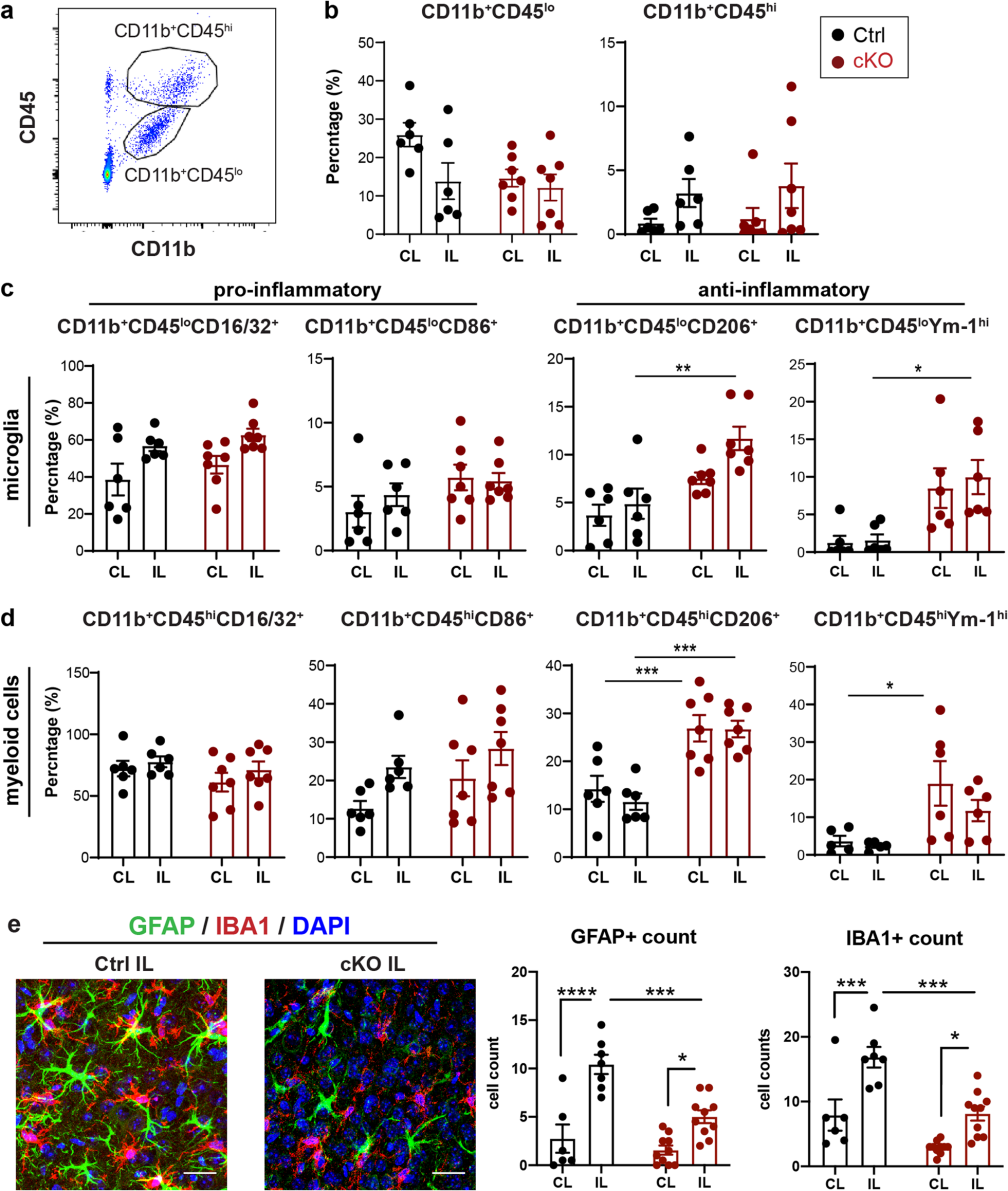
Selective deletion of microglial *Nhe1* reduced inflammatory responses in cKO mice at 3 days post-TBI. **a** Representative gating strategy of CD11b^+^CD45^lo^ microglia and CD11b^+^CD45^hi^ myeloid cells using flow cytometry. **b** Percentages of the CD11b^+^CD45^lo^ microglia and CD11b^+^CD45^hi^ macrophage populations within live singlet cells of Ctrl or cKO brains at 3 days post-TBI. **c**, **d** Expressions of pro- and anti-inflammatory markers within CD11b^+^CD45^lo^ or CD11b^+^CD45^hi^ populations. Data are mean ± SEM, N = 6 for Ctrl (3 males, 3 females) and N = 7 for cKO (4 male, 3 females). **e** Representative images and quantification of GFAP^+^ reactive astrocytes and IBA1^+^ microglia/macrophages in the peri-lesion cortex of Ctrl or cKO brains at 3 days post-TBI. Scale bar = 20 µm. Data are mean ± SEM, *N* = 6 for Ctrl (3 males, 3 females) and *N* = 10 for cKO (6 males, 4 females). * *p* < 0.05, ** *p* < 0.01, *** *p* < 0.001, **** *p* < 0.0001

To understand the underlying mechanisms of the increased oligodendrogenesis in the post-TBI cKO brains, we examined the profiling of microglia and infiltrated myeloid cells in Ctrl or *Nhe1* cKO brains at 3 days post-TBI with flow cytometry (Fig. 3a). No difference in the percentage of microglial cells (CD11b^+^CD45^lo^) and infiltrated myeloid cells (CD11b^+^CD45^hi^) were detected in CL or IL hemispheres of the Ctrl and *Nhe1* cKO mice (Fig. 3b, *p* > 0.05). Further probing of the expression of pro-inflammatory markers CD16/32 and CD86 did not show any differences between Ctrl and cKO microglia and/or myeloid cells (Fig. 3c, d). However, the percentage of anti-inflammatory CD206-positive microglia/myeloid cells and Ym-1^hi^-positive microglia were significantly increased in the IL hemisphere of the cKO brains, compared to the Ctrl (Fig. 3c, d, *p* < 0.05). Further characterization of microglial cells or reactive astrocytes in the peri-lesion cortex of Ctrl or cKO brains by immunofluorescent staining revealed that TBI induced significant increases in GFAP^+^ astrocytes and IBA1^+^ microglia/macrophage counts in both the Ctrl and cKO brains at 3 days post-TBI (Fig. 3e, *p* < 0.05), but, the cKO brains showed significantly attenuated GFAP^+^ astrocyte and IBA^+^ microglia/macrophage counts were significantly attenuated in the peri-lesion area of the cKO brains, compared to the Ctrl (Fig. 3e, *p* < 0.001). Taken together, these findings demonstrate that selective deletion of microglial *Nhe1* gene promotes the restorative activation of microglia/myeloid cells and reduces astrogliosis in the cKO brains.

### Selective deletion of microglial *Nhe1* altered inflammation-related transcriptome profile in microglia/macrophages after TBI

**Fig. 4:**
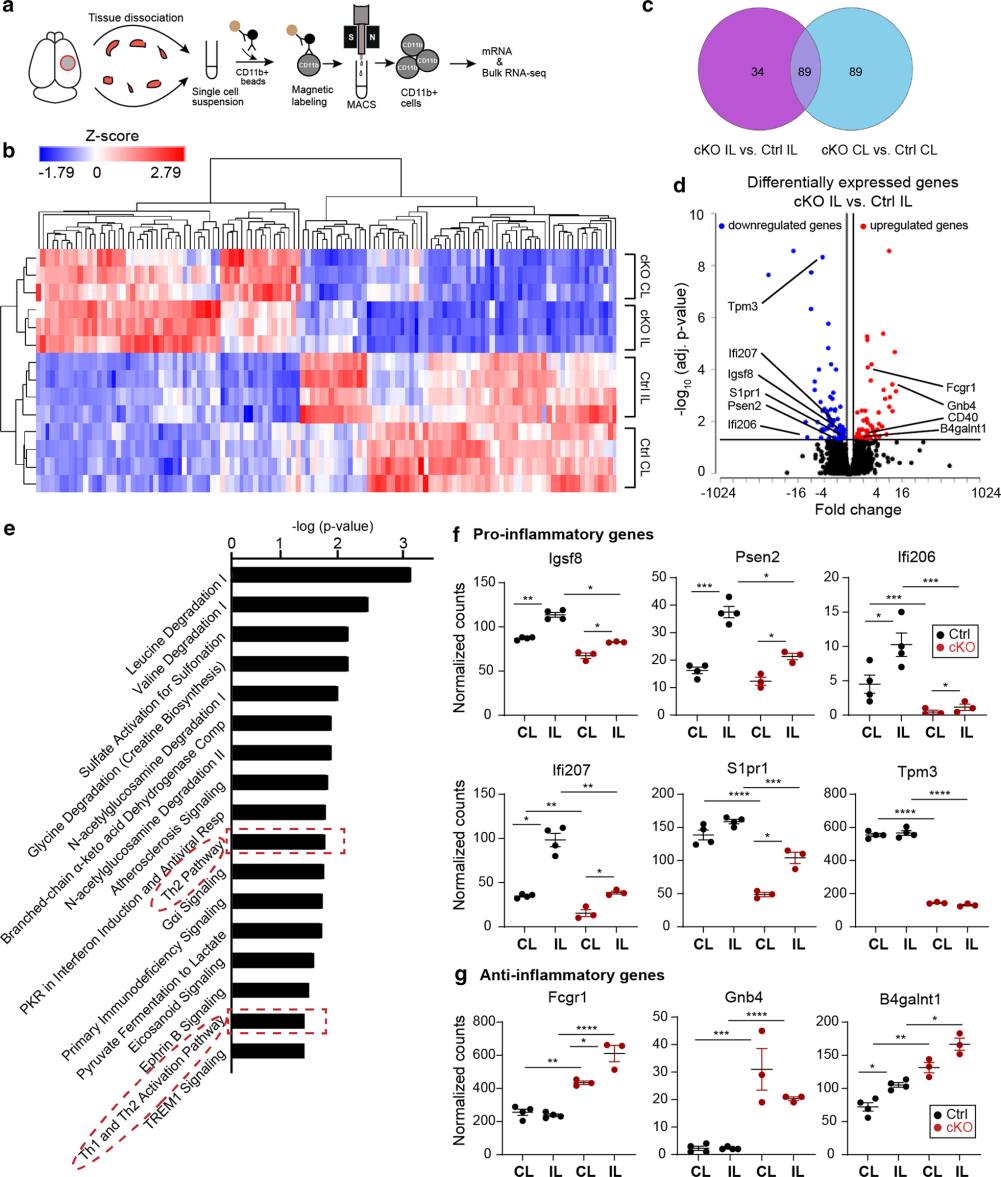
Microglial *Nhe1* deletion alters pro- and anti-inflammatory transcriptome profiles in microglia/macrophages at 3 days post-TBI. **a** Bulk RNAseq of CD11b^+^ microglia/myeloid cells isolated from CL and IL hemispheres of Ctrl and cKO mice at 3 days post-TBI. **b** Unsupervised hierarchical clustering and heatmap illustration of up- and down-regulated genes. **c** Venn diagram depicting differential gene expression; FDR q value ≤ 0.05, fold change of ≥ 1.2 or ≤ -1.2. **d** Volcano plots illustrate the gene expression pattern (detected with log2 fold change of ≥ 1.2 and FDR *q*-value ≤ 0.05). **e** Enrichment analysis showing significantly altered top canonical pathways using Ingenuity Pathway Analysis software. **f**,** g** Scatter plots showing expression of Th1/Th2 pathway and pro- and anti-inflammatory genes presented as normalized counts. Data are mean ± SEM, *N* = 4 for Ctrl (all males) and *N* = 3 for cKO (all males). *p < 0.05, **p < 0.01, ***p < 0.001, ****p < 0.0001

To understand how deletion of microglial *Nhe1* affects microglial restorative function, we performed bulk RNA sequencing (RNA-seq) of CD11b^+^ cells isolated from the CL and IL hemispheres of Ctrl and *Nhe1* cKO brains at 3 days post-TBI (Fig. 4a). Unsupervised hierarchical clustering analysis demonstrated clear separation between CL and IL hemispheres of cKO and Ctrl brains (Fig. 4b). 123 differentially expressed genes (DEGs) were identified in the IL hemispheres of cKO mice, compared to Ctrl mice (fold change ≥ 1.2 or ≤ -1.2 and FDR *q*-value ≤ 0.05, Fig. 4c); among those, 56 genes were upregulated and 67 downregulated (Fig. 4d). Ingenuity Pathway Analysis (IPA) showed significantly altered enrichment pathways, including Th1 and Th2 activation pathways (Fig. 4e, *p* < 0.05), which are known to regulate inflammation states in microglia and macrophages by secreting a variety of cytokines/factors, such as IFN-γ, IL-4, IL-10, TGF-β, and osteopontin [25, 26]. Within these pathways, multiple pro-inflammatory genes were triggered by TBI in the Ctrl microglia/macrophages, but significantly decreased in the cKO microglia/macrophages, such as *Psen2*, *Ifi206*, *Ifi207*, and *Igsf8* (Fig. 4d, f), all reported to be involved in microglia/macrophage-mediated inflammation [27–30]. *S1pr1* and *Tpm3* genes are also involved in inflammation and expression of these two genes were reduced significantly in cKO IL hemisphere compared to ctrl IL hemisphere (Fig. 4f). Additionally, the cKO microglia/macrophages showed elevated expressions of *Fcgr1*, *Gnb4*, and *B4galnt1* genes which stimulate anti-inflammatory activation [29, 31]. On the other hand, the non-lesion CL hemispheres displayed 178 DEGs between Ctrl and cKO microglia/macrophages (Fig. 4c, Additional file 1: Fig. S4a), with IPA analysis showing significantly altered IL-8 signaling pathway with reduced inflammatory genes such as *Napepld*, *Vcam1*, *Rnd1* [32] in the cKO microglia/macrophages (Additional file 1: Fig. S4b, c). Change of selected key pathway genes have been validated by qRT-PCR (Additional file 1: Fig. S5). Additionally, changes of pathways in naïve Ctrl and cKO brains (Additional file 1: Fig. S6), as well as pathway networks specific to CL or IL hemispheres of Ctrl and cKO mice after TBI have been analyzed using Metascape (https://metascape.org) (Additional file 1: Fig. S7a-b). We also probed for myelination-related genes reported in literature [33–36], however, no significant differences were detected (Additional file 1: Fig. S7c). Taken together, our bioinformatic analysis reveals that deletion of microglial *Nhe1* attenuates the expression of inflammation-related transcriptomes, but stimulates restorative microglial activation transcriptome profiles after TBI.

### Post-TBI administration of selective NHE1 inhibitor HOE642 accelerated neurological function recovery

**Fig. 5:**
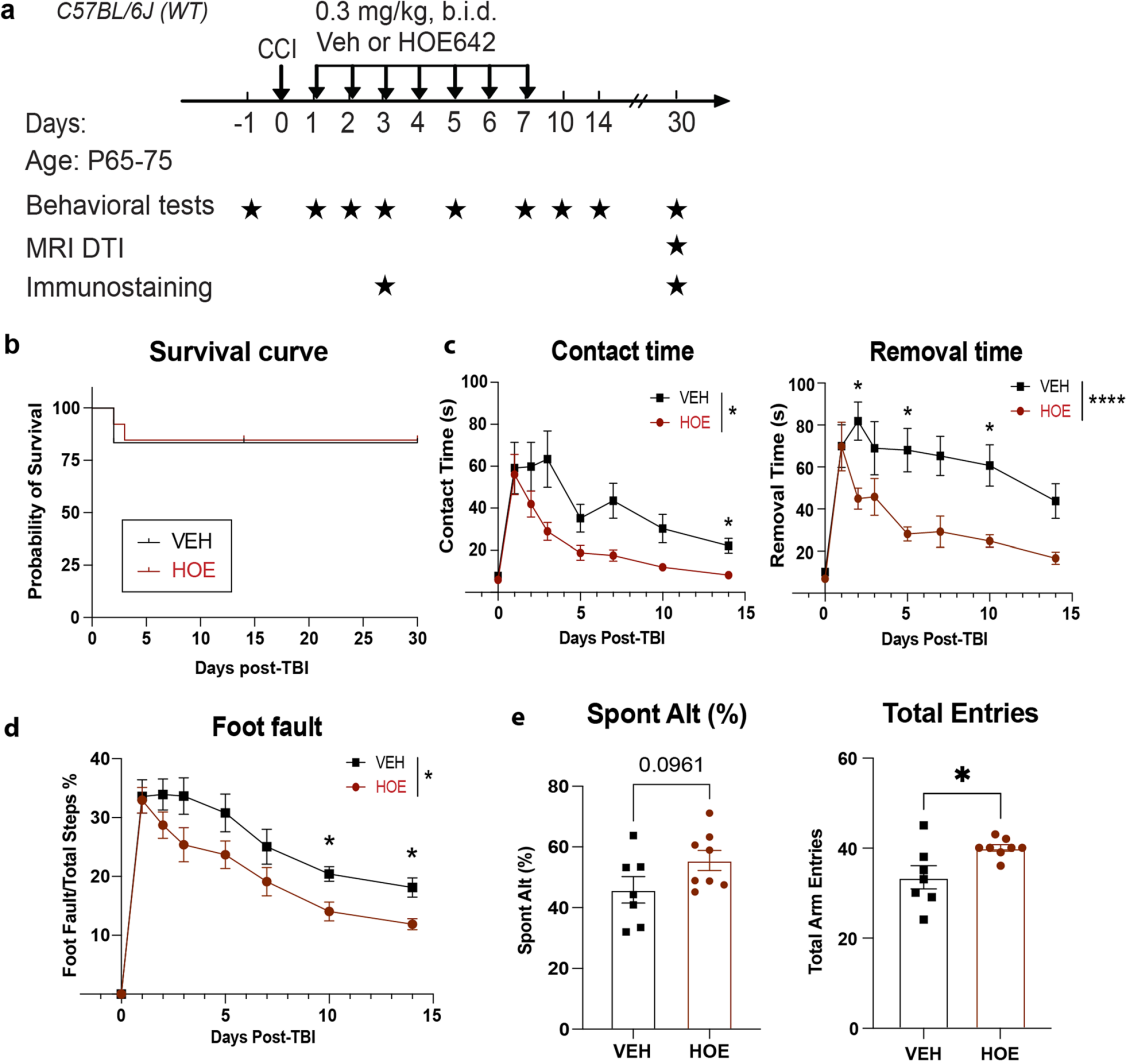
Post-TBI administration of selective NHE1 inhibitor HOE642 in C57BL/6 wild-type mice accelerated neurological function recovery. **a** Experimental protocol. Either Veh (2.5% DMSO in PBS) or HOE642 (0.15 mg/kg) was administered twice per day from 1 to 7 days post-TBI in the C57BL/6J wild-type (WT) mice. **b** Survival rate of Veh or HOE-treated mice at 1–30 days post-TBI. **c** Adhesive contact and removal time of Veh and HOE-treated mice at 1–14 days after TBI. **d** Foot fault test of the same cohort of mice in C. **e** Y-maze test of the same cohort of mice in C at 30 days post-TBI. *N* = 7–11. Data are mean ± SEM. *p < 0.05, ****p < 0.0001, Veh. vs. HOE-treated groups

We next explored the efficacy of targeting NHE1 protein with a pharmacological inhibition approach in reducing the TBI-induced functional deficits. Figure 5a illustrated our administration protocol of Veh (DMSO) or a potent NHE1 inhibitor HOE642 (0.3 mg/kg body weight/day, twice per day, i.p.) at 24 h post-TBI. Compared to the Veh-treated TBI mice, HOE642 administration did not affect mortality during 30 days post-TBI (Fig. 5b). However, the HOE642-treated TBI mice exhibited significant improvements in sensorimotor function (adhesive contact/removal test and foot fault test) during the 14 days post-TBI recovery period (Fig. 5c, d, *p* < 0.05). In assessing the same cohort of mice in cognitive function with Y-maze test at 30 days post-TBI, the HOE642-treated mice exhibited an improved trend of performance in spontaneous alternation rate than the Veh-treated mice (Fig. 5e, *p* = 0.09), indicating improved working memory function [37]. These HOE-treated TBI mice also showed significantly increased locomotor activity reflected by their total arm entries (Fig. 5e, p < 0.05). These outcomes are consistent with the *Nhe1* cKO mice shown in Fig. 1.

### Characterization of the HOE642-mediated protective effects in TBI mice

**Fig. 6:**
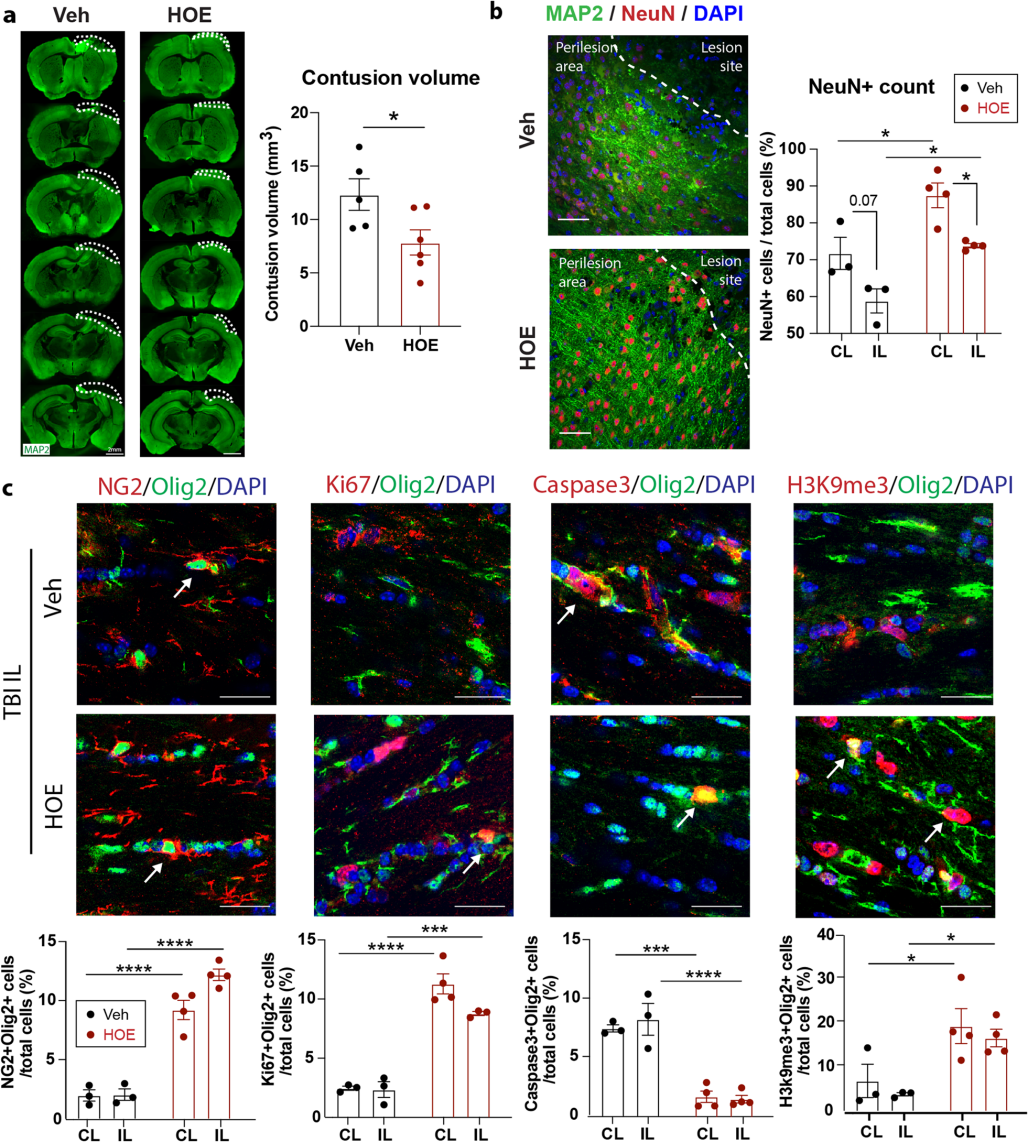
Post-TBI administration of selective NHE1 inhibitor HOE642 in C57BL/6 wild-type mice showed neuroprotection and enhanced oligodendrogenesis. **a** Contusion volume of Veh or HOE-treated brains at 3 days post-TBI by MAP2 staining. Data are mean ± SEM. *N* = 5–6. **b** Immunostaining of MAP2 and NeuN in the peri-lesion cortex of Veh or HOE-treated brains at 3 days post-TBI. Scale bar = 50 µm. **c** Representative images and quantification of Olig2 colocalized with NG2, Ki67, Caspase-3, and H3K9me3 in CC at 3 days post-TBI. Arrows: colocalized cells. Scale bar = 10 µm. Data are mean ± SEM. *N* = 3–4. *p < 0.05, ***p < 0.001, ****p < 0.0001

Compared to the Veh-treated mice, the HOE-treated mice exhibited significantly smaller contusion volume and increased NeuN^+^ neurons in both CL and IL peri-lesion cortex at 3 days post-TBI (Fig. 6a, b, *p* < 0.05). Preservation of hippocampal structures and higher NeuN^+^ cell counts in CA1, CA3 regions were also detected in the cKO TBI mice (Additional file 1: Fig. S8), indicating that post-TBI administration of HOE642 has neuroprotective effects. In line with the enhanced white matter repair detected in the *Nhe1* cKO mice**,** the HOE-treated TBI brains displayed significantly increased NG2^+^Olig2^+^ OPCs (*p* < 0.0001), Ki67^+^Olig2^+^ proliferative OLs (*p* < 0.001), reduced Caspase3^+^Olig2^+^ apoptotic OLs (*p* < 0.0001), as well as H3K9me3^+^Olig2^+^ differentiating OLs in the CC white matter tracts, compared to the Veh-treated brains at 3 days post-TBI (Fig. 6c, *p* < 0.05). Moreover, flow cytometry of CD11b^+^CD45^lo^ microglia and CD11b^+^CD45^hi^ myeloid cells from the Veh or HOE-treated TBI brains (Additional file 1: Fig. S9a, b) showed that the anti-inflammatory phenotype (CD206^+^) of microglia cells were selectively increased in the IL hemisphere of the HOE-treated TBI brains (Additional file 1: Fig. S9c, *p* < 0.05), while sparing the pro-inflammatory phenotypes of myeloid cells or microglial cells (Additional file 1: Fig. S9c, d). Immunostaining further confirmed that IBA1^+^ microglia/macrophages and GFAP^+^ reactive astrocytes were significantly reduced in the peri-lesion cortex of HOE-treated TBI brains (Additional file 1: Fig. S9e, *p* < 0.0001). These findings collectively suggest that post-TBI administration of NHE1 protein inhibitor HOE642 protected neurons and reduced inflammatory responses and gliosis in the brain, which can concertedly contribute to the improved functional outcome post-TBI.

### Long-term effects of NHE1 protein blockade on white matter integrity after TBI

**Fig. 7:**
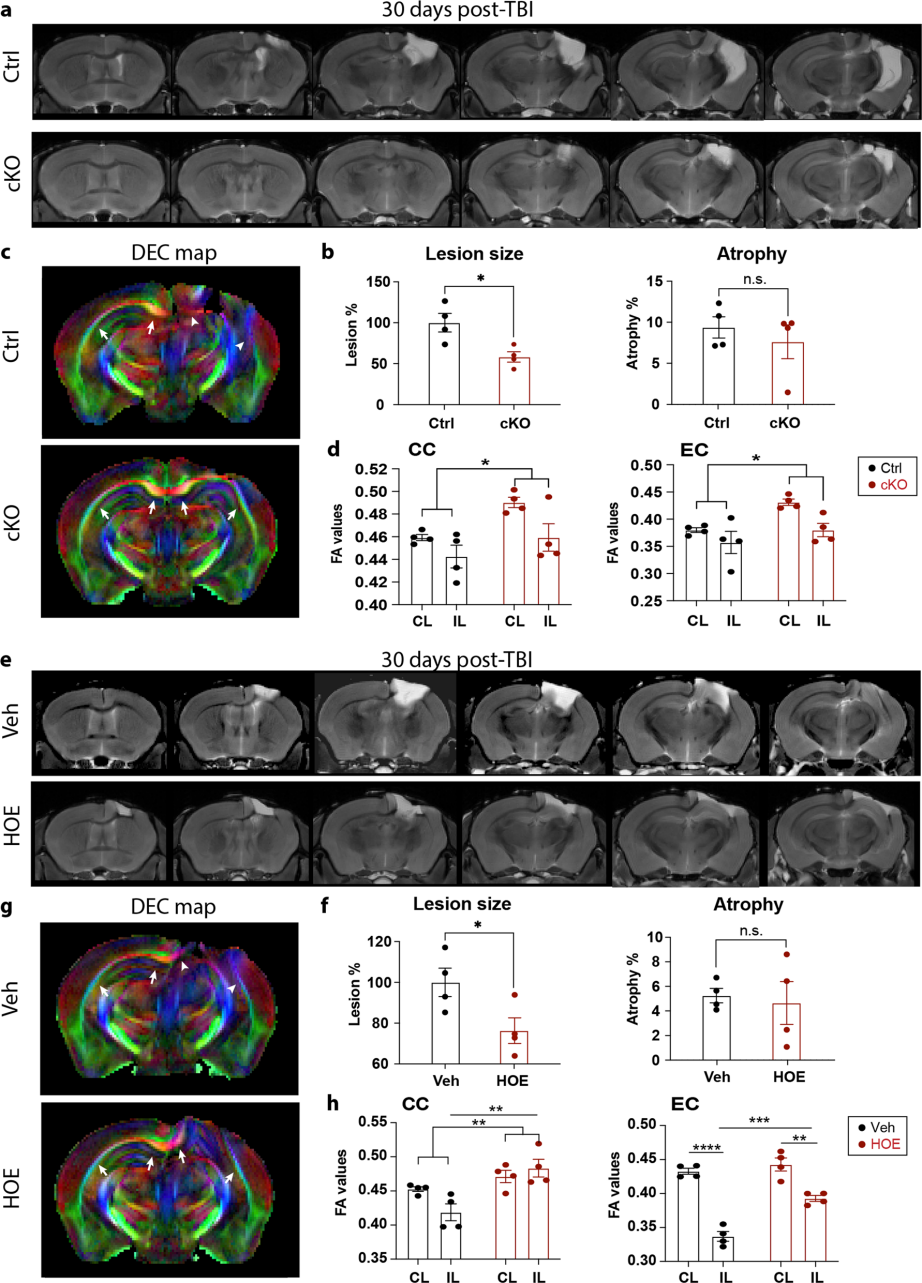
Increased white matter integrity in microglial *Nhe1* cKO and HOE642-treated mice at 30 days post-TBI. **a**, **b** Representative images of brains from rostral to caudal, and calculations of lesion volume and atrophy of T2 MRI of the ex vivo brains of Ctrl and cKO mice at 30 days post-TBI. **c**, **d** Representative DTI diffusion encoded color (DEC) map and analysis of FA in the white matter tracts (CC and EC) of the same cohort of mice in **a**. Colors hues represent the principle orientation of the diffusion tensor (red = left/right, blue = dorsal/ventral, and green = rostral/caudal) with the intensity weighted by the FA. **e**, **f** Representative images and calculations of lesion volume and atrophy of T2 MRI of the ex vivo brains of the Veh-control and HOE642-treated mice at 30 days post-TBI. **g-h** Representative DTI DEC map and analysis of FA in the white matter tracts of the same cohort of mice in **e**. Data are mean ± SEM. *N* = 4. *p < 0.05, **p < 0.01, ***p < 0.001, ****p < 0.0001

We further assessed whether the *Nhe1* cKO brains and HOE-treated brains exhibited long-lasting preservation of white matter integrity by 30 days post-TBI via MRI DTI of the ex vivo brains of the same cohort of mice after completing neurological function testing. The cKO mice exhibited significantly reduced brain lesion volume at 30 days post-TBI, compared to the Ctrl mice (Fig. 7a, b). Interestingly, both the *Nhe1* cKO brains and HOE-treated brains displayed increased FA values in the CC and EC white matter tracts (of both hemispheres) at 30 days post-TBI, compared to either Ctrl or Veh-treated TBI brains (Fig. 7c, p < 0.05). It has been reported that white matter demyelination is associated with higher radial diffusivity (RD) and medial diffusivity (MD), but not necessarily with reduced axial diffusivity (AD) [38, 39]. We detected significantly lowered RD and MD in the EC of the HOE-treated TBI brains, and higher AD in the EC of *Nhe1* cKO TBI brains (Additional file 1: Fig. S10), but, no significant differences in RD, MD, or AD were detected in the CC (Additional file 1: Fig. S10). Taken together, these data collectively demonstrate that blocking microglial NHE1 protein has sustained long-term protective effects on white matter myelination, and reveals NHE1 protein as a potential therapeutic target for white matter repair after TBI.

## Discussion

TBI triggers potent neuroinflammatory reactions in the brain, mediated by a complex cascade of cellular and molecular events [40], including excessive glutamate release, rapid depletion of ATP, increased oxidative stress, and elevated inflammation, altogether contributing to the loss of myelin sheath, OLs death, and inhibition of OL maturation after TBI [1, 7, 41]. Within minutes after TBI, microglia become activated, releasing proinflammatory cytokines (IFN-γ, TNF-α, IL-1β), reactive oxygen species (ROS), and inflammasome (such as NLRP3)-containing extracellular vesicles, etc., which exacerbate injury [42–44]. However, microglia are highly plastic cells and can also respond to cytokines released by Th2 cells in the surrounding environment, such as IL-4 and IL-13, to promote an alternative state phenotype that is associated with growth and tissue repair [40]. Thus, the alternatively activated microglial cells can provide neuroprotective qualities, such as oligodendrogenesis, angiogenesis, and remyelination, following injury post-TBI [44]. Regulating microglial phenotypic conversion to minimize the neurotoxic events, while preserving the neuroprotective qualities, emerges as a novel strategy in developing new treatments for minimizing white matter damage and promoting remyelination in TBI patients.

**Fig. 8:**
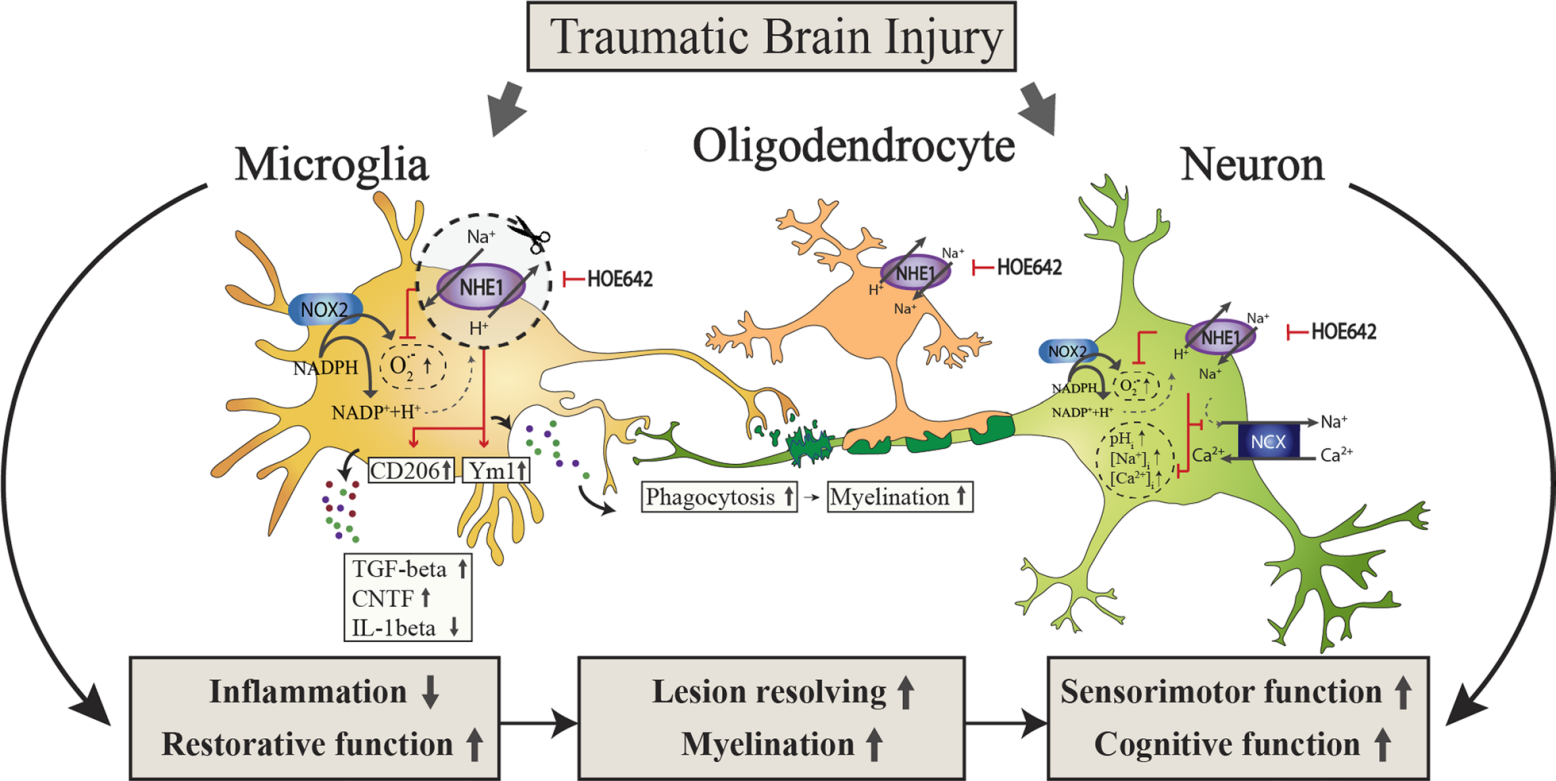
Schematic illustration of microglial NHE1 protein activation in brain tissue damage/repair after TBI. TBI triggers NHE1 activation in both microglia and neurons. Specific inhibition of NHE1 protein in microglia not only reduced acute contusion volume and neurodegeneration, but also significantly ameliorated inflammatory microenvironment, promoting restorative microglial function and myelination after TBI. These outcomes were recapitulated with administration of a potent NHE1 inhibitor HOE642 post-TBI. Taken together, these findings demonstrated that blocking NHE1 protein stimulates restorative microglial activation in neuroprotection and oligodendrogenesis, which contributes to accelerated brain repair and neurological function recovery after TBI

We previously reported that NHE1 protein, which mediates H^+^ efflux in exchange of Na^+^ influx, is essential in regulating microglial homeostatic pH_i_ [13], similar to the roles of the ATPase H^+^ pump in myelin-supporting microglia [9], and the voltage-gated H^+^ channel Hv1 [45]. Upon brain injury, NHE1 protein is rapidly activated and mediates H^+^ extrusion activity to maintain the optimal alkaline microglial pH_i_ for NADPH oxidase (NOX) activation, ROS production, and inflammation (Fig. 8) [13, 14]. In the current study, blocking NHE1 protein activity, either by genetic deletion or pharmacological inhibition, reduced the microglial pro-inflammatory phenotype and increased the anti-inflammatory profiling during the acute phase after TBI (Fig. 8) where early activation of the restorative phenotype is proven to be essential in long-term tissue repair and functional recovery after TBI [42]. Comparisons of TBI-induced signaling pathways to naïve brains revealed consistently activated pathways in the cKO brains under both naïve condition and in non-lesion and lesion hemispheres, such as the ID1 (inhibitor of DNA binding) signaling pathway, eNOS (endothelial NOS) signaling, and Hif1α signaling (Additional file 1: Fig. S6c). Among the top changed genes within these pathways, we identified significantly elevated *Hif1a*, master gene for glucose metabolism [46], *Slc2a5* (encoding GLUT5), mediating glucose/fructose transportation [47], and a 20-fold decrease of *Ldhb*, which convert lactate to pyruvate, all involved in glucose metabolism pathways. Indeed, NHE1 blockade leads to more acidic pH_i_, which is linked to stimulating the master transcription regulator *Hif1a* [46]. Future studies are warranted for further investigation of these signaling pathway changes in regulation of energy metabolism and brain repair. Moreover, the cKO brains showed significantly increased percentage of NeuN^+^ neurons (Fig. 1d), without significant improvement for lesion volume or absolute NeuN^+^ counts (Fig. 1c, d, Additional file 1: Fig. S11a). On the other hand, compared to the Veh-treated brains, HOE642 treatment significantly increased both absolute cell counts and percentage of NeuN^+^ cells in the peri-lesion area (Additional file 1: Fig. S11b). The observed neuroprotection (cerebral cortical and hippocampal) in the cKO and HOE642-treated mice likely results in their accelerated neurological function recovery after TBI.

Microglia play an important role in supporting normal myelinogenesis during development and adulthood, with distinctly high expression of a lysosomal marker LAMP2, as well as upregulated genes such as *Spp1, Gpnmb, Igf1*, as well as the ATPase H^+^ transporting V0 Subunit D2 gene *Atp6v0d2* [9]. The transition of microglial functions from detrimental to restorative is essential in the initiation of OPC differentiation and the start of remyelination in a demyelination model [48] as well as in stroke [49]. In both microglia-specific *Nhe1* cKO mice and HOE642-treated WT mice post-TBI, we detected increased anti-inflammatory phenotype of microglia and enhanced white matter myelination and oligodendrogenesis (Fig. 8). This is corroborated by our RNAseq analysis which revealed significantly reduced proinflammatory pathways in the cKO microglia, compared to the Ctrl microglia. These changes could concertedly enhance the proliferation and differentiation of the OL/OPCs, and result in improved white matter repair, which synergistically contributes to the accelerated neurological functional recovery after TBI.

In the current study, we used a drug administration regimen from 1 to 7 days post-TBI for the NHE1 specific inhibitor HOE642 and observed pronounced beneficial effects in these mice. We believe that these outcomes directly result from blocking NHE1 activity and attenuating cellular H^+^ extrusion. It has been reported that TBI patients with a high cerebral lactate/pyruvate ratio at 1–4 days post-TBI displayed worsened outcomes [50]. In a mouse CCI-induced TBI study [45], investigators detected sustained peri-lesion brain tissue acidosis (pH_e_ at 6.6–6.7) for up to 28 days post-TBI and an accumulation of intracellular H^+^ in CD11b^+^/CD45^+^ cells [45]. In addition, selective deletion of Hv1 in transgenic mice attenuated H^+^ extrusion as well as NOX2 protein expression in CD11b^+^/CD45^+^ microglial/macrophage cells after TBI [45]. We did not detect a significant difference in the *Hvcn1* gene expression (encoding Hv1 protein) in our RNAseq of Ctrl and cKO CD11b^+^ microglial cells with or without TBI (Additional file 1: Fig. S7d). Taken together, these findings suggest that TBI-induced dysregulation of pH homeostasis plays an important role in the development of neuroinflammation and neurological function deficits. H^+^ regulatory proteins (such as NHE1 and Hv1) emerge as potential therapeutic targets for TBI.

Our study has several limitations. As NHE1 protein is ubiquitously expressed in all cell types in the CNS, the current pharmacological approach is not cell-specific and can affect NHE1 protein function in all cell types. Since pH_i_ is involved in regulating OL division and their differentiation into mature OLs [51], blocking oligodendrocytic NHE1 via HOE642 could also regulate pHi in OLs, and have a direct impact on oligodendrogenesis in post-TBI white matter repair. Further studies with cell-selective deletion of Nhe1 in OLs are warranted to elucidate specific roles of NHE1 protein in OLs. Moreover, compared to WT controls, OL clusters with increased ERK/MAPK and SPP1 transcriptome in single cell RNAseq data were detected from the microglial *Nhe1* cKO white matter brain tissues after stroke (data not shown), which further support role of microglial NHE1 protein in microglia–OL communications. In addition, HOE642 treatment may block astrocytic NHE1 protein and indirectly modulate microglial function through astrocyte–microglia interactions [52, 53]. It was reported that loss of NHE1 protein enhances neuronal excitability and leads to increased occurrence of epilepsy [54]. However, we detected neuroprotective effects and significant reduction of contusion volume in the HOE642-treated mice. Thus, future studies are warranted to dissect the protective effects of HOE642 in various cell types after TBI. In addition, since sex-specific differences in male and female microglial functions have been observed after brain injury [55], we disaggregated the data to evaluate for sex-dependent differences after TBI. We observed similar trends of data between males and females in Ctrl or cKO mice (Additional file 1: Fig. S12), thus we only tested in males for the pharmacological study. Further study is warranted utilizing both males and females in evaluating the efficacy of HOE642 treatment after TBI. Lastly, the current in vivo study does not directly investigate roles of microglial NHE1 activity in regulation of oligodendrocyte function after TBI. Future in vitro studies with additional approaches (such as interactions of co-cultured microglia and oligodendrocytes, or characterization of changes of secretomes of microglial cells) shall further shed light on our in vivo study findings.

## Conclusion

To date, no treatment was proven effective for reducing white matter damage and/or stimulating white matter remyelination after TBI. Our findings demonstrate that blockade of NHE1 protein activity, either by genetic deletion or pharmacological approach, accelerated post-TBI neurological functional recovery by reducing microglial inflammatory activation, neurodegeneration, and stimulating oligodendrogenesis in white matter repair (Fig. 8). We identified NHE1 protein as a potential therapeutic target for post-TBI brain repair and neurological functional improvement.

## Supplementary Information

**Additional file 1:** Supplementary Information.

## Data Availability

All the data associated with this study are present in the paper or the Supplementary Materials. The RNA sequencing data have been deposited to the Gene Expression Omnibus database with experiment series accession number GSE199869.

## Abbreviations

ARRIVE: Animal Research Reporting In Vivo Experiments
AD: Axial diffusivity
CL: Contralateral
Ctrl: Control
CC: Corpus callosum
DEG: Differentially expressed gene
DTI: Diffusion tensor imaging
EC: External capsules
FA: Fractional anisotropy
GEO: Gene Expression Omnibus
IPA: Ingenuity Pathway Analysis
IL: Ipsilateral
pH_i_: Intracellular pH
MACS: Magnetic-activated cell sorting
MD: Medial diffusivity
cKO: Conditional knockout
MBP: Myelin basic protein
NHE1: Na^+^/H^+^ exchanger isoform-1
NOX: NADPH oxidase
OL: Oligodendrocytes
OPC: Oligodendrocyte progenitor cells
PFA: Paraformaldehyde
RD: Radial diffusivity
RNA-seq: RNA sequencing
TBI: Traumatic brain injury
WT: Wild-type
