# Supplementary material for: Microglial–oligodendrocyte interactions in myelination and neurological function recovery after traumatic brain injury
Source: J Neuroinflammation. 2022 Oct 5;19:246. doi: 10.1186/s12974-022-02608-6 (PMC9533529; doi:10.1186/s12974-022-02608-6)
Supplement: Supplementary file 1 — Additional file 1: Supplementary Information. [file 12974_2022_2608_MOESM1_ESM.docx]

**Microglial-oligodendrocyte interactions in myelination and neurological function recovery after traumatic brain injury**

*Shanshan Song^1,2,3*^, Md Nabiul Hasan^1,2,3*^, Lauren Yu^1,2^, Satya S. Paruchuri^1,2^, John P. Bielanin^1,2^, Shamseldin Metwally^1,2^, Helena C.M. Oft^1,2^, Sydney G. Fischer^1,2^, Victoria M. Fiesler^1,2,3^, Tanusree Sen^4^, Rajaneesh K. Gupta^4^, Lesley M. Foley^5^, T. Kevin Hitchens^5,6^, C. Edward Dixon^3,4^, Franca Cambi^1,2,3^, Nilkantha Sen^4^, Dandan Sun^1,2,3#^*

**Supplementary Information**

**Material and Methods**

***Animals***

All animal studies were approved by the University of Pittsburgh Medical Center Institutional Animal Care and Use Committee, which adhere to the National Institutes of Health Guide for the Care and Use of Laboratory Animals and reported in accordance with the Animal Research: Reporting In Vivo Experiments (ARRIVE) guidelines (1). Animals were provided with food and water ad libitum and maintained in a temperature-controlled environment in a 12/12 h light-dark cycle. All efforts were made to minimize animal suffering and the number of animals used.

*Cx3cr1-CreER^+/-^* control (Ctrl) mice and *Cx3cr1-CreER^+/-^;Nhe1^f/f^* (*Nhe1* cKO) mice were established as described previously (2). Both genotypes of mice (male or female) at postnatal day 30-40 (P30-40) received tamoxifen (Tam, Sigma) (75 mg/kg body weight/day at a concentration of 20 mg/ml in corn oil, intraperitoneally) for 5 consecutive days. Considering the 21-day self-renewing cycles in the *Cx3cr1^+^* peripheral infiltrating bone marrow-derived myeloid cells (BMDM) (3), to minimize the effects on *Cx3cr1^+^* BMDMs, a 30-day post-injection waiting period (**Fig. 1a**) was used for clearance of Tam (4-6) and for replenishing of *Cx3cr1*^+^ monocytes (7) prior to sham or traumatic brain injury (TBI) procedures, which achieved a complete replenishment of *Cx3cr1^+^* BMDM (7) and has been proven to be effective in our study (2, 8) as well as in others (9-11). Surgeries and all outcome assessments were performed by investigators blinded to mouse genotypes and experimental group assignments.

For the inhibitor study, 2-3 months old, male C57BL/6J wild-type (WT) mice were used. A potent NHE1 protein inhibitor HOE642 (cariporide, Sigma-Aldrich, USA) was dissolved at 1 mg/ml in DMSO as stock solution, and diluted to 0.025 mg/ml in PBS immediately before injection. 2.5% DMSO in PBS was used as vehicle control (Veh). Veh or HOE642 (0.3 mg/kg body weight/day, i.p., b.i.d.) was administered daily from 24 h until 7 days post-TBI. Since the half-life of HOE642 is about 3.5 h, and its inhibitory effect on platelets declined to 50 % at 4 h post-administration (12), data at 1 d post-stroke were collected at least 4 h post-initial injection.

***Controlled cortical impact*** ***(CCI)-induced TBI procedures***

Adult mice (male and female, 2-3 months old) were kept under 1.5% isoflurane anesthesia during the procedure and the core temperature (37.0 °C) was maintained by a small animal temperature controller pad throughout all procedures. Controlled cortical impact (CCI) was performed by placing the mice in a stereotaxic frame (Ambient Instruments), and a 3.5 mm craniectomy (via an electric drill) was made in the right parietal bone midway between bregma and lambda with the medial edge lateral to the midline, leaving the dura intact. Mice were impacted at 4.5 m/s with a 20 ms dwell time and 1.2 mm depression using a 3-mm-diameter convex tip, mimicking a moderate TBI (13). The mice were allowed to recover under a heating lamp during a 30 min recovery period. Sham control animals underwent the identical craniectomy procedures with an electric drill without the impact.

***Behavioral function tests***

Neurological functional deficits in mice were screened in a blinded manner with the following tests: adhesive contact test, adhesive removal test, foot fault test, and y-maze spontaneous alternation test, all considered reliable for identifying and quantifying sensorimotor or cognition deficits in mouse models of TBI (14-16).

1. ***Adhesive Test.*** The adhesive contact test and the adhesive removal test were used to measure somatosensory deficits as described previously (17). Animals were tested prior to TBI and at 1, 2, 3, 5, 7, 10, and 14 days after TBI. Two pieces of adhesive tape (4 mm × 3 mm) were attached to the forepaws in an alternating sequence and with equal pressure by the experimenter before each trial. The contact time was defined as the time at which the animal first made contact with the tape, and the removal time was defined as the time at which the animal removed the tape. The trial ended after the adhesive patch was removed or after 2 min had elapsed. Pre-operative training was carried twice per day for consecutive three days.
2. ***Foot fault test.*** Each mouse was placed on a stainless-steel grid floor (20 cm × 40 cm with a mesh size of 4 cm^2^) elevated 1 m above the floor, prior to TBI and at 1, 2, 3, 5, 7, 10, and 14 days after TBI. The animals were first be habituated to the grid floor for 1 min and then tested for three 1-min trials. Data were expressed as the number of foot fault errors made by the forelimbs contralateral to the injury hemisphere as a percentage of total steps.

***Y-maze test:*** The Y-maze spontaneous alternation test was used to assess spatial working memory, as described previously (18). Each animal was placed into one arm of the Y-maze and monitored over an 8-min duration with an overhead video tracking system at 30 days post-TBI. A sequential list of arms entries was analyzed using an automated Sequence Analysis Tool macro in excel. Spontaneous alternation was only counted when a mouse entered three different arms consecutively. The spontaneous alternation % was calculated as the percentage of the number of triad spontaneous alternation in total arm entries minus two.

***MRI and DTI of ex vivo brains***

At 30 days post-TBI, the same cohort of mice from the behavioral assessments were euthanized with CO_2_ overdose, transcardially perfused with 0.1 M PBS (pH 7.4), followed by ice-cold 4 % paraformaldehyde (PFA) in 0.1 M PBS, and decapitated, as described before (2) . Brains were maintained within the skull to avoid anatomical deformation. After post-fixation in 4% PFA overnight, heads were stored in PBS solution at 4°C. Magnetic Resonance Imaging (MRI) was performed at 500MHz using a Bruker AV3HD 11.7 T/89 mm vertical bore small animal MRI scanner, equipped with a 20-mm quadrature radiofrequency (RF) coil and Paravision 6.01 software (Bruker Biospin). Following positioning and pilot scans, T2-weighted images (T2WI) were acquired using a Rapid Acquisition with Relaxation Enhancement (RARE) sequence, with the following parameters: Time of Echo / Time of Repetition (TE/TR) = 20/3500ms, averages = 2, 256 x 256 matrix, 25 slices with a 1 mm slice thickness, a RARE factor = 4, and a field of view (FOV) of 22 x 22 mm. A Diffusion Tensor Imaging (DTI) data set covering the entire brain was collected using a multislice spin echo sequence with 3 reference and 30 non-collinear diffusion-weighted images with the following parameters: TE/TR = 22/5000 ms, 4 averages, matrix size = 192 × 192 reconstructed to 256 × 256, field of view = 22 × 22 mm, 25 axial slices, slice thickness = 1 mm, b-value = 1200 s/mm^2^, and Δ/δ􏰀= 10/5 ms. DTI and T2 datasets were analyzed with DSI Studio (http://dsi-studio.labsolver.org/). In a blinded manner, region of interests (ROIs) were drawn segmenting corpus callosum (CC) and external capsule (EC) in both the contralateral (CL) and ipsilateral (IL) hemispheres from 4 scanned sections in each brain. Values of fractional anisotropy (FA) were calculated for each ROI, as described before (2).

***Flow cytometry***

Mice were euthanized with overdose of CO_2_ and transcardially perfused with ice-cold saline, as described before (2). After removal of cerebellum and meninges, CL and IL hemispheric tissues were separated and dissociated into single cell suspensions using a neural tissue dissociation kit with the gentleMAC Octo Dissociator (Miltenyi Biotech Inc., Germany). Myelin was removed using the 30/70 Percoll gradient method as described (2). Cells were stained with BUV395-CD11b (BD Biosciences, USA), BV510-CD45 (BioLegend, USA), PE-Ym1 (Abcam, USA), eFluor 450-CD16/32 (Thermo Fisher Scientific, USA), PE-CY7-CD206 (Thermo Fisher Scientific, USA), Alexa Fluor 700-CD86 (BD Bioscience, USA) antibodies for 20 min at 4^o^C in the dark. Samples were acquired using an LSRII flow cytometer (BD Biosciences, USA) equipped with FACS Diva software. Data were analyzed using the Flow Jo (Tree Star Inc, USA) software.

***Microglia isolation***

Single cell suspensions from the CL and IL hemispheric tissues were collected at 3 days post-TBI as described above, and CD11b^+^ microglia/macrophages were isolated by magnetic-activated cell sorting (MACS) using the CD11b MicroBeads (Miltenyi Biotech, USA). Briefly, the single cell suspensions were incubated with the CD11b MicroBeads for 20 min at 4^o^C, before washing through an MS column (Miltenyi Biotech Inc., Germany) placed on a OctoMACS magnetic field separator (Miltenyi Biotech Inc., Germany), with the magnetically labeled CD11b^+^ cells maintained in the column and unlabeled CD11b^-^ cells washed away. The isolated CD11b^+^ cells were subsequently used for RNA sequencing and bioinformatic analysis.

***Bulk RNA sequencing and bioinformatics analysis***

Bulk RNA sequencing and bioinformatic analysis was performed in the CD11b^+^ microglia/macrophage samples sorted by MACS, as described above. Total RNA was extracted using Direct-zol RNA MicroPrep kit (Zymo Research, #R2060). Library was prepared using a Smart Seq v4 library prep kit (Takara Bio) and sequenced on an Illumina Novaseq 6000 platform with 40 million reads per sample counted. STAR aligner was used to map the raw reads with a mouse genome (mm10) with default setting and differential gene expression analysis was performed using the GSA package of Partek Flow 8.0 software (Partek, USA). Genes with a false discovery rate (FDR) ≤ 0.05 and fold change (FC) ≥ 1.2 or ≤ 1.2 were identified as differentially expressed genes (DEGs). Ingenuity Pathway Analysis (IPA) software (QIAGEN Redwood City, www.qiagen.com/ingenuity) was used for the enrichment analysis of the DEGs to identify significantly enriched biological pathways using Fisher’s exact test. Additional pathway enrichment and network analysis were conducted using Metascape (<https://metascape.org>). The identified transcriptome profiles were subsequently verified by real-time qPCR (described below). The sequencing data have been deposited to the Gene Expression Omnibus (GEO) database with experiment series accession number GSE199869.

***Quantitative real-time PCR***

CD11b^+^ cells (0.5 x 10^6^ Cells/sample) isolated from TBI brains were lysed in Trizol lysis buffer immediately following MACS sorting, and RNA was extracted using the Direct-Zol RNA MicroPrep Kit (Zymo Research) according to the manufacturer's instructions. ND-1000 (NanoDrop) spectrophotometer was used to quantify RNA. RNA was converted to cDNA by reverse transcription using the iScript Reverse Transcription Supermix (Bio-Rad) according to the manufacturer’s protocol. Quantitative RT-PCR was performed using iTaq Universal SYBR Green Supermix (Bio-Rad) on a CFX 96 Touch Real-Time PCR Detection System. Relative gene expression analyses were performed using the 2−ΔΔCt method with triplicate reactions for each gene evaluated. Primer sequences used are listed in **Table S1**.

***Immunofluorescent staining***

Mice were euthanized with overdose of CO_2_ and transcardially perfused with 0.1 M PBS (pH 7.4), followed by ice-cold 4 % PFA in 0.1 M PBS as described before (2) . Brains were cryoprotected with 30% sucrose after an overnight post-fixation in 4% PFA (2). Coronal sections (25 μm, at the level of 0.26 mm anterior to bregma) were selected and processed for immunofluorescent staining. The sections were incubated with blocking solution (10% normal goat serum and 0.3% Triton X-100 in PBS) for 1 h at room temperature and were then incubated with the following antibodies for overnight at 4°C: mouse monoclonal anti-GFAP (1:200, Cell Signaling Technology) and rabbit polyclonal anti-IBA1 (1:200, Wako), mouse monoclonal anti-MAP2 (1:200, EMD Millipore) and rabbit monoclonal anti-NeuN (1:200, Abcam), mouse monoclonal anti-APC (1:200, EMD Millipore) and rabbit polyclonal anti-MBP (1:200, Abcam), mouse monoclonal anti-Olig2 (1:200, Millipore) and rabbit polyclonal anti-NG2 (1:200, Millipore) or rabbit polyclonal anti-Ki67 (1:200, Millipore) or rabbit polyclonal anti-caspase3 (1:200, Cell Signaling Technology) or rabbit polyclonal anti-H3K9me3 (1:200, Abcam). After washing in TBS-Triton X-100 (0.3%) for 3×10 min, the sections were incubated with goat anti-mouse Alexa 488-conjuagated IgG (1:200, Invitrogen) and goat anti-rabbit Alexa 546-conjugated IgG (1:200, Invitrogen) in the blocking solution for 1 h. For negative controls, brain sections were stained with the secondary antibodies only. After washing for 3 times, nuclei were stained with DAPI (1:1000, Thermo Fisher Scientific) for 15 min at room temperature. Sections were mounted with Vectashield mounting medium (Vector Laboratories). At least 3 fluorescent images were captured for each area under 40× lens using a Nikon A1R confocal microscope (Nikon, Japan). Identical digital imaging acquisition parameters were used and images were obtained and analyzed in a blinded manner throughout the study.

***Statistical analysis***

Unbiased study design and analyses were used in all the experiments. Blinding of investigators to experimental groups were maintained until data were fully analyzed whenever possible. Power analysis were performed based on the mean and variability of data from our laboratory. N=6 mice/group for behavioral tests, immunostaining, flow cytometry, qPCR, and N=4 for RNAseq and MRI/DTI were sufficient to give us 80% power to detect 10% changes with 0.05 one-sided significance. Data were expressed as mean ± SEM and all data were tested for normal distribution. Not normally distributed data were analyzed by Mann-Whitney U test or other appropriate alternative tests according to the data (Prism, GraphPad, USA). Two-tailed Student’s t-test 95% confidence was used when comparing two conditions. For more than two conditions, two-way ANOVA analysis was used. P value < 0.05 was considered statistically significant (Prism, GraphPad, USA).


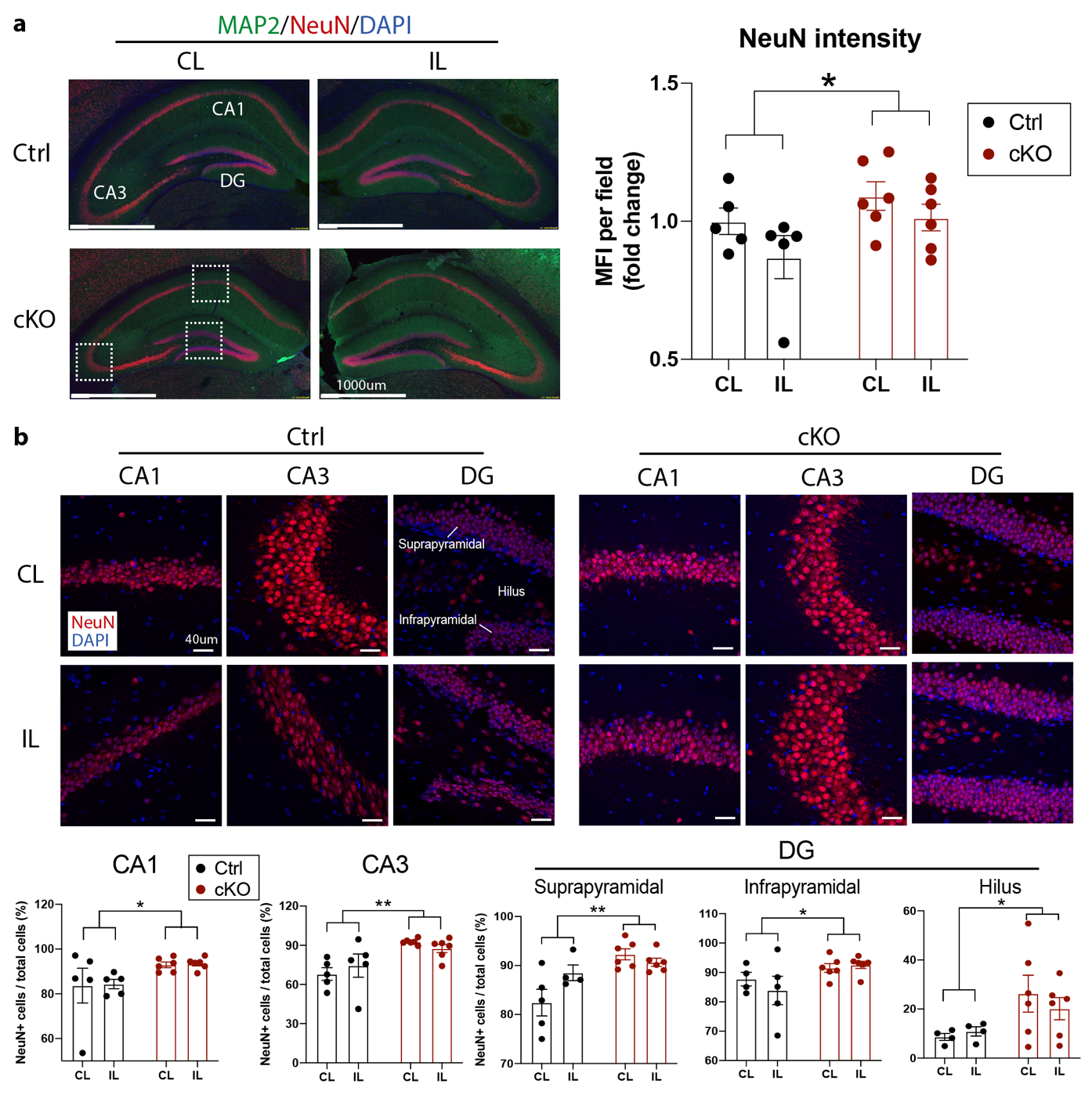


**Fig. S1. Reduced TBI-mediated hippocampal neurodegeneration in *Nhe1* cKO mice.**

**a.** Representative 4x images of neuronal markers MAP2 and NeuN expressions and analysis of NeuN intensity in the CL and IL hippocampus of the same cohort of Ctrl and cKO mice as in **Fig. 1**. Scale bar = 1000 µm. **b.** Representative 40x images and NeuN^+^ cell counts in CA1, CA3, and dentate gyrus. Scale bar = 40 µm. * p < 0.05; ** p < 0.01.

**
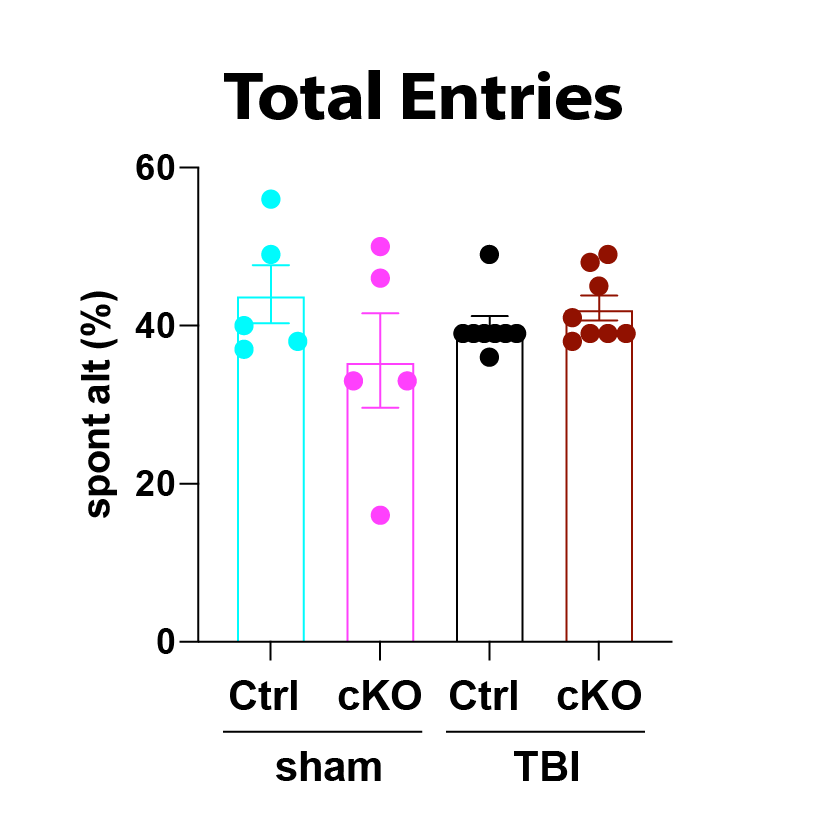
**

**Fig. S2. Microglial *Nhe1* deletion did not change locomotor activities at 30 days after TBI.**

Total entries from the Y-maze test in the same cohort of mice as in Fig. 1e. Data are mean ± SEM. N = 8.


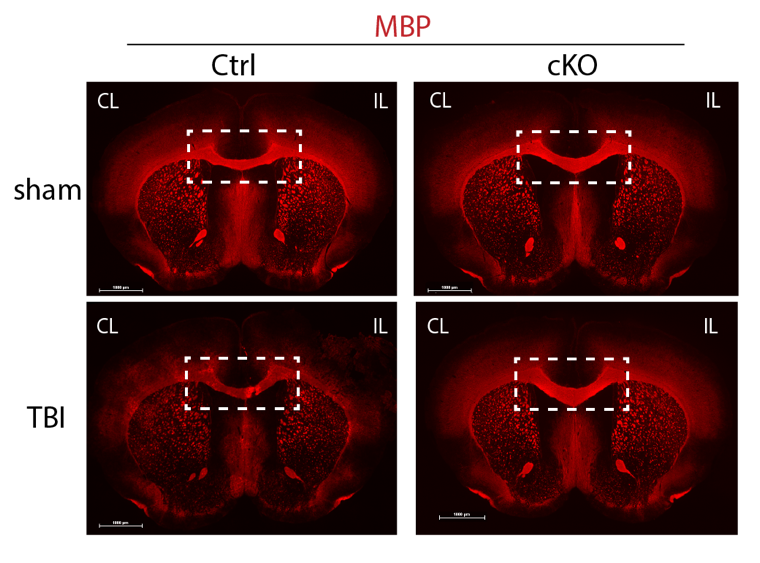


**Fig. S3. Microglial *Nhe1* cKO mice exhibited increased corpus callosum (CC) thickness.**

Whole brain images of MBP fluorescent staining at 1 day after sham or CCI procedures. Boxed areas are shown in Fig. 2a.


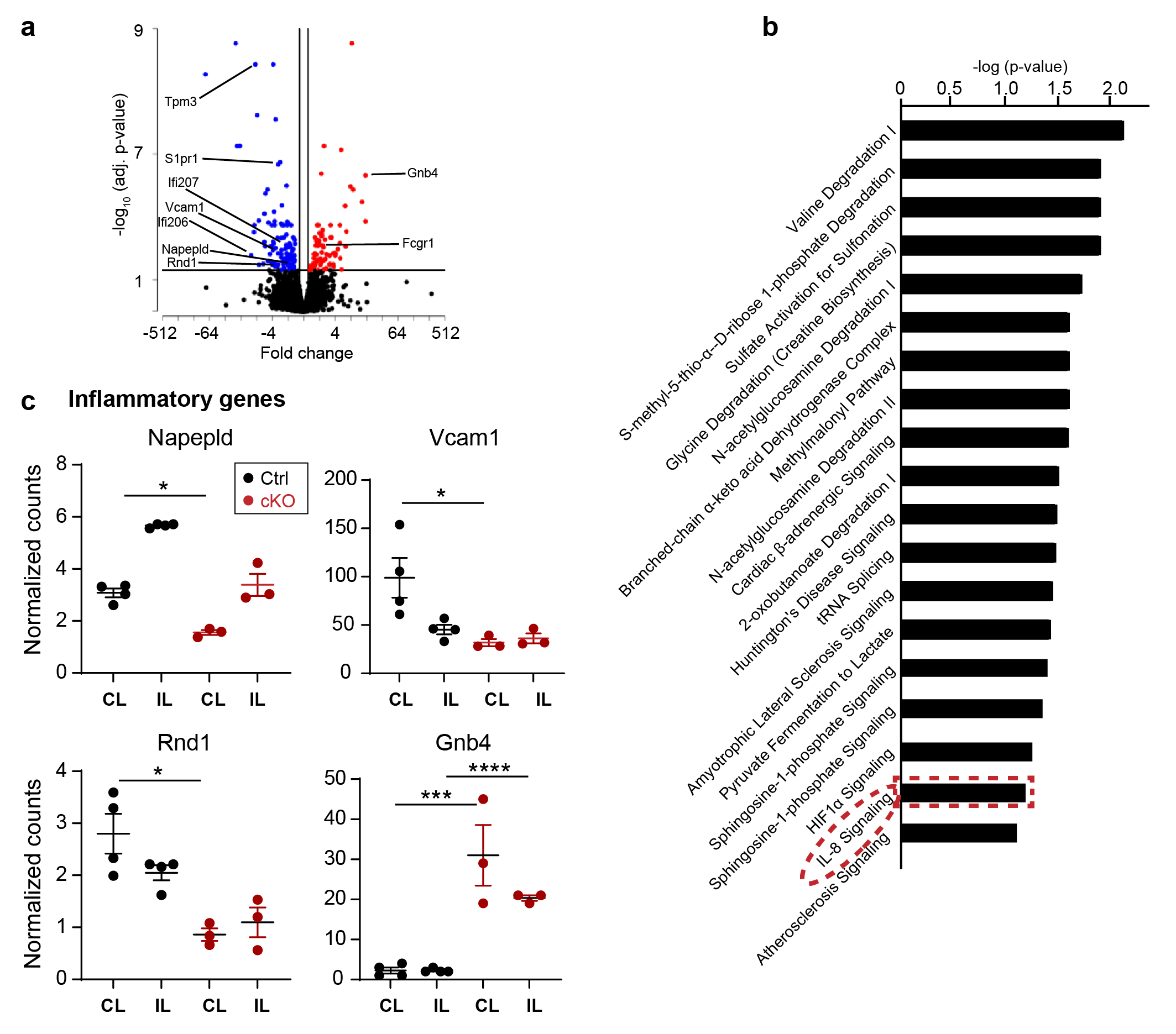


**Fig. S4. RNA-seq transcriptome analysis of CD11b^+^ microglial cells after TBI.**

**a.** Volcano plots illustrate the gene expression pattern detected (log2 fold change ≥1.2 and FDR q-value ≤ 0.05) from the same dataset in Fig. 4. **b.** Enrichment analysis showing significantly altered top canonical pathways using Ingenuity Pathway Analysis software. **c.** Scatter plots showing expression of IL-8 signaling genes presented as normalized counts. Data are mean ± SEM. N = 4 for Ctrl (all male) and N = 3 for cKO (all male). *p < 0.05, ***p < 0.01, ****p < 0.0001.

**
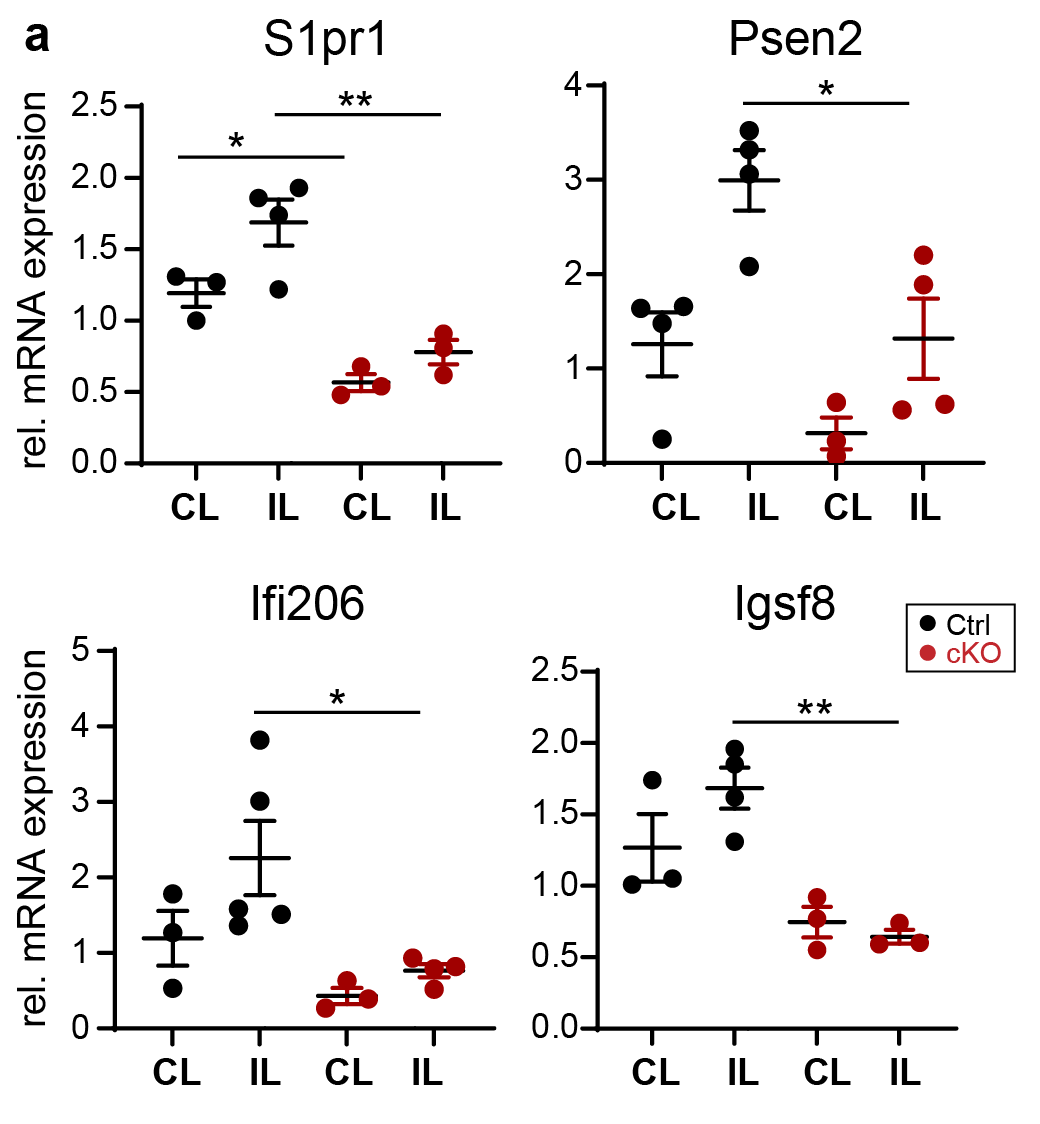
**

**Fig. S5. qRT-PCR validation of genes showing reduced microglial expression of inflammatory genes in cKO mice at 3 days post-TBI.**

**a.** qRT-PCR quantification of *S1pr1, Psen2*, *Ifi206 and Igsf8,* genes from CD11b^+^ microglia/myeloid cells isolated from CL and IL hemispheres of Ctrl and cKO mice at 3 days post-TBI. Data are mean ± SEM. N = 5 for Ctrl (2 female, 3 male) and N = 4 for cKO (2 female, 2 male). * p < 0.05, ** p < 0.01.


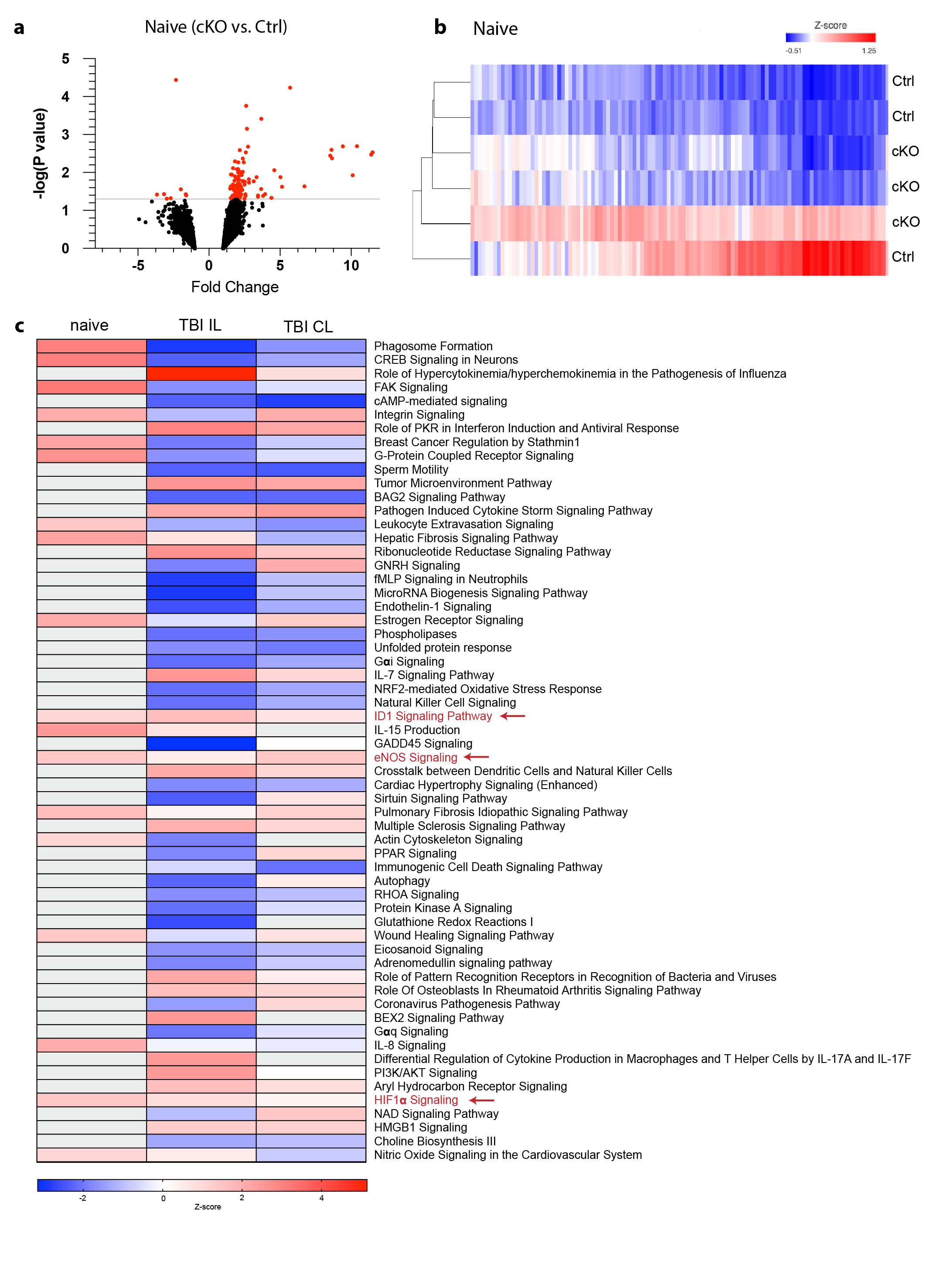


**Fig. S6. CD11b^+^ microglial cells from Ctrl and cKO mice displayed differentially regulated signaling pathways under naïve and post-TBI conditions.**

**a.** Volcano plot illustrating the gene expression pattern (detected with log2 fold change of ≥1.2 and FDR q-value ≤ 0.05) in the naïve brains of Ctrl and cKO mice. N=3. **b.** Unsupervised hierarchical clustering and heatmap illustration of up- or down-regulated genes in naïve brains of Ctrl and cKO mice. **c.** Heatmap comparing pathway changes in naïve brains and TBI (CL and IL hemispheres) of Ctrl and cKO mice. TBI data are from the same cohort in **Fig. 4**.


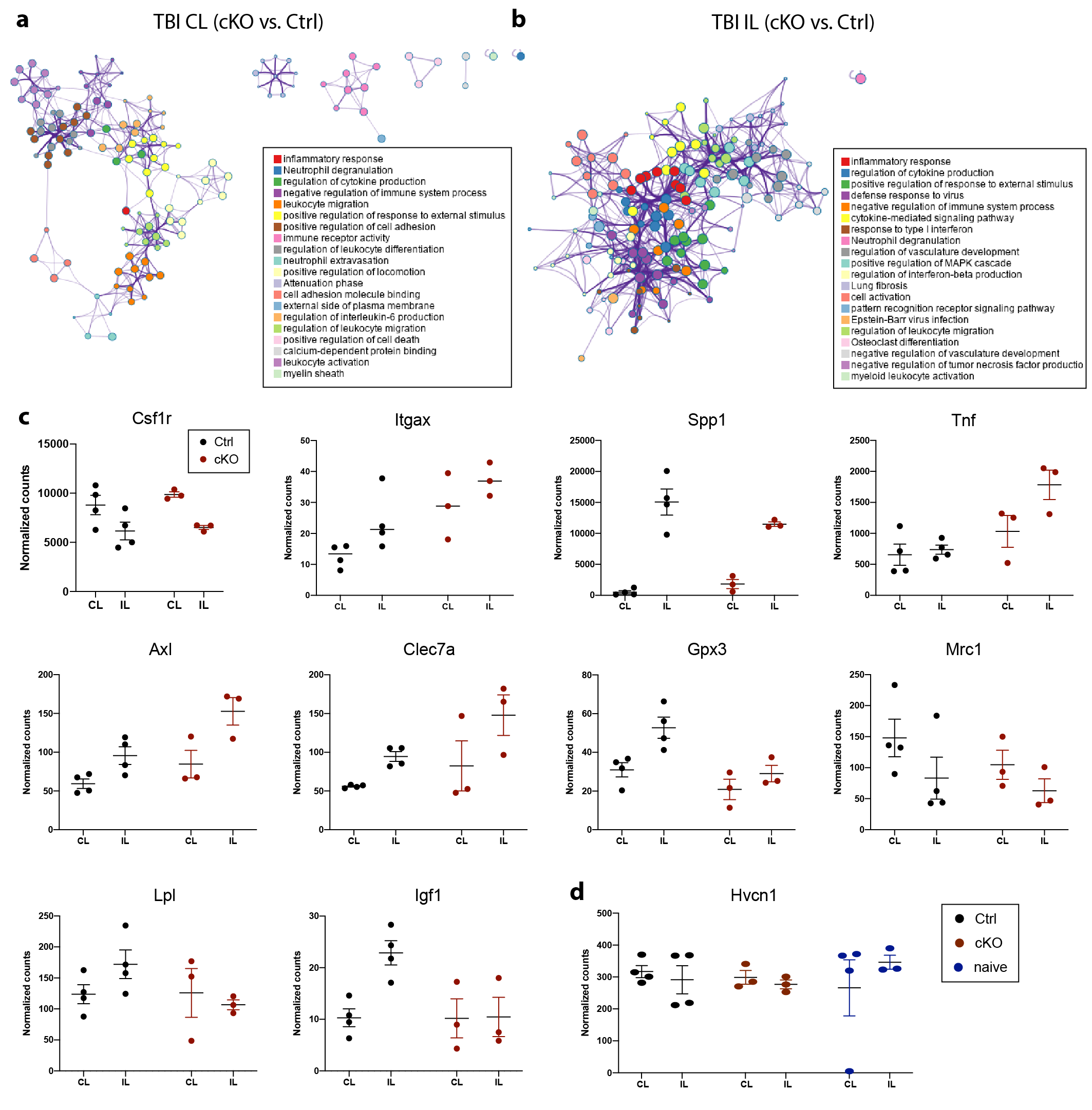


**Fig. S7. Differentially regulated pathways and myelination-related genes in Ctrl and cKO brains after TBI**

**a-b.** Metascape enrichment and network analysis of the DEGs from CL or IL hemispheres cKO TBI mice compared to the Ctrl TBI mice, from the same dataset in **Fig. 4**. Each node represents a term associated with an ontological cluster (in same color), with larger nodes representing terms with more input genes. Terms with a similarity score > 0.3 are connected by lines whose weight corresponds to the degree of similarity. **c.** Myelination-related genes reported in literature CL and IL hemispheres of Ctrl and cKO mice after TBI. **d.** Hvcn1 gene expression in naïve and Ctrl or cKO brains post-TBI.


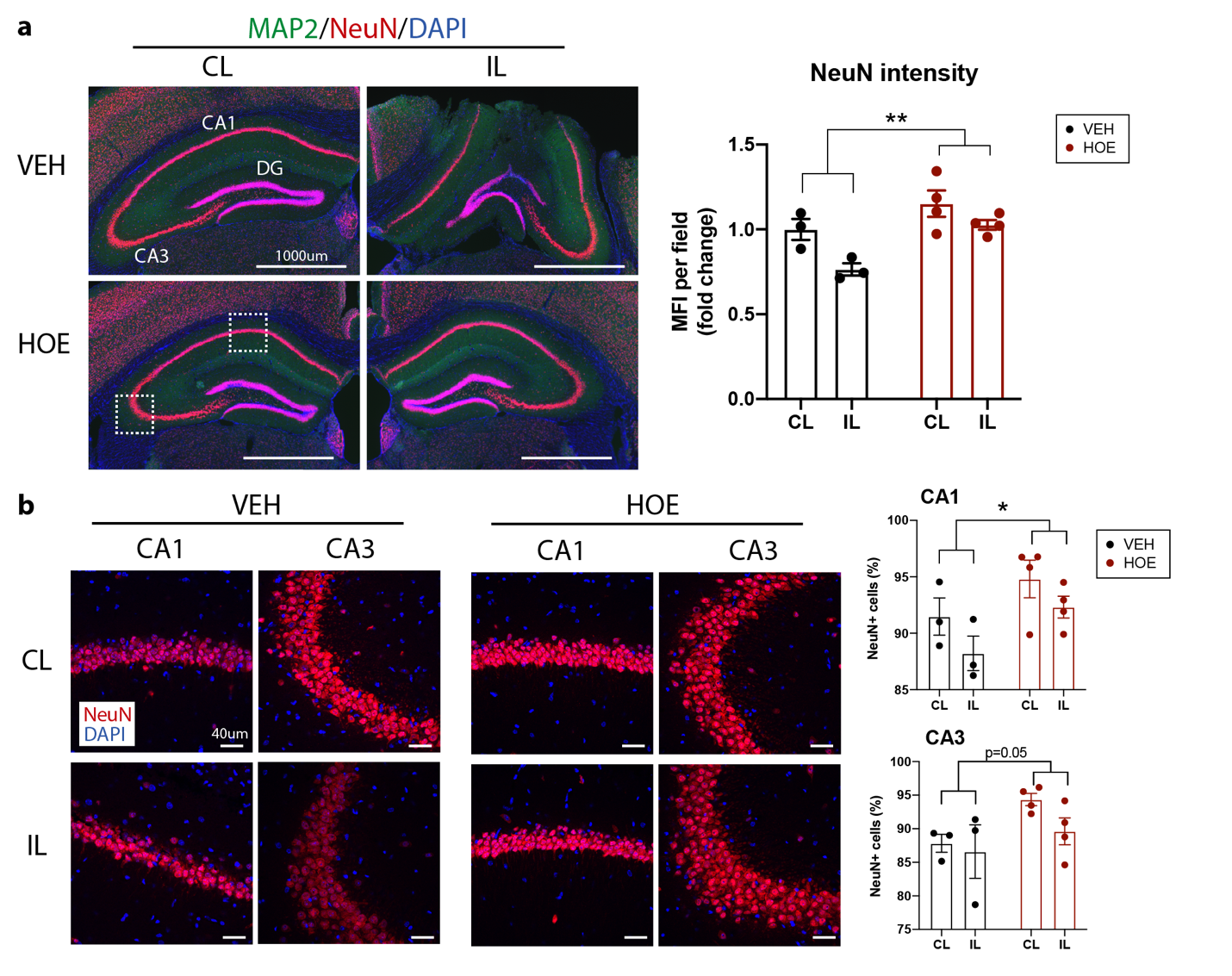


**Fig. S8. HOE642 administration after TBI protects hippocampal neurons.**

**a.** Representative 4x images of neuronal markers MAP2 and NeuN expressions and analysis of NeuN intensity in the CL and IL hippocampus in the same cohort of mice as in **Fig. 6**. Scale bar = 1000 µm. **b.** Representative 40x images and NeuN^+^ cell counts in CA1 and CA3 in the CL and IL hippocampus in the same cohort mice as in **Fig. 6**. Scale bar = 40 µm. * p < 0.05, ** p < 0.01.


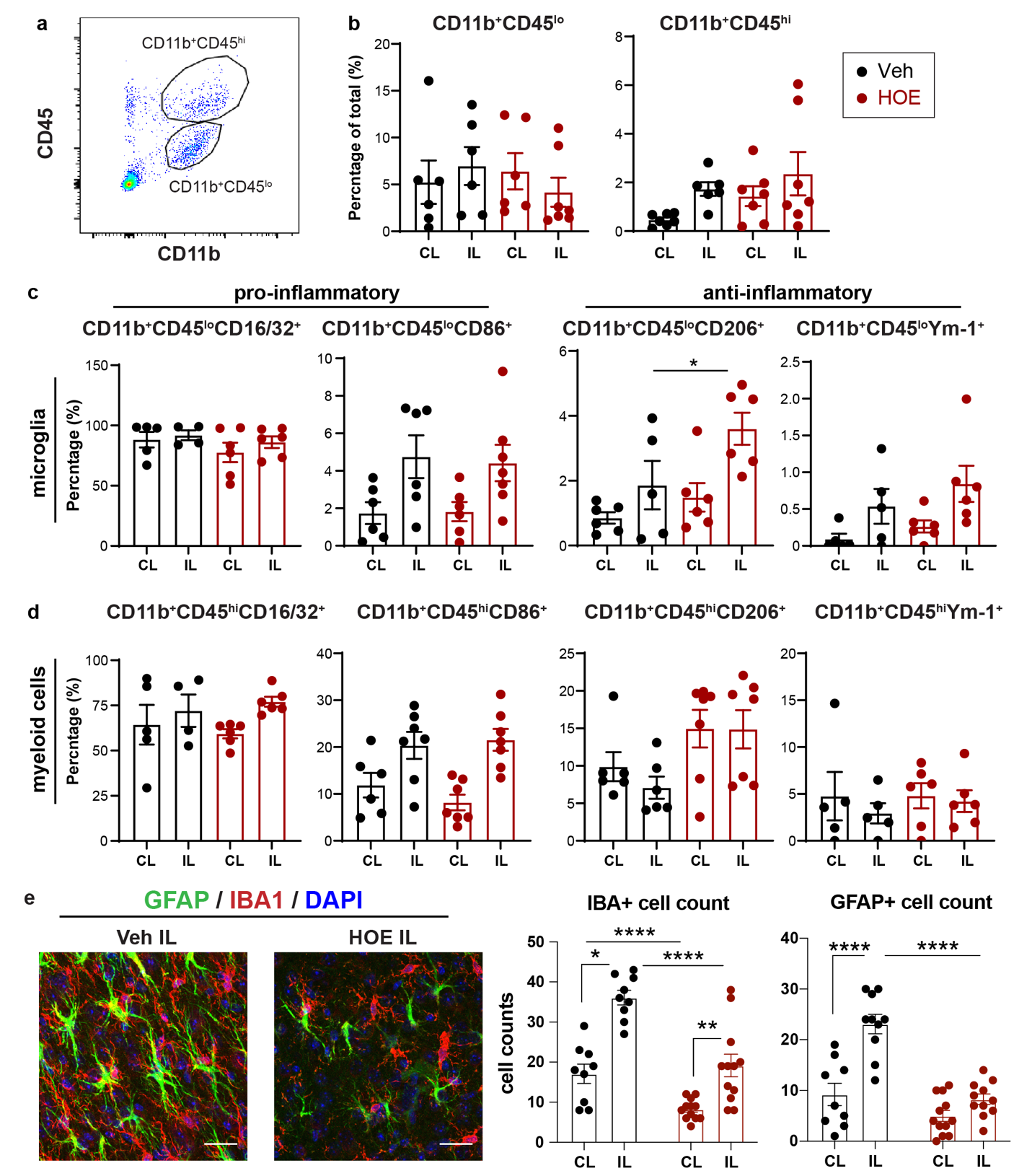


**Fig. S9. Post-TBI administration of selective NHE1 inhibitor HOE642 in C57BL/6 wild-type mice reduced inflammatory responses.**

**a.** Representative gating strategy of CD11b^+^CD45^lo^ microglia and CD11b^+^CD45^hi^ myeloid cells using flow cytometry. **b.** Percentages of the CD11b^+^CD45^lo^ microglia and CD11b^+^CD45^hi^ macrophage populations within live singlet cells of Veh or HOE-treated brains at 3 days post-TBI. **c-d.** Expressions of pro- and anti-inflammatory markers within CD11b^+^CD45^lo^ or CD11b^+^CD45^hi^ populations. Data are mean ± SEM, N = 6 for vehicle (all male) and N = 7 for HOE (all male). **e.** Representative images and quantification of GFAP^+^ reactive astrocytes and IBA1^+^ microglia/macrophages in the peri-lesion cortex of Veh or HOE-treated brains at 3 days post-TBI. Scale bar = 20 µm. Data are mean ± SEM, N = 9-12. * p < 0.05, **** p < 0.0001.

**
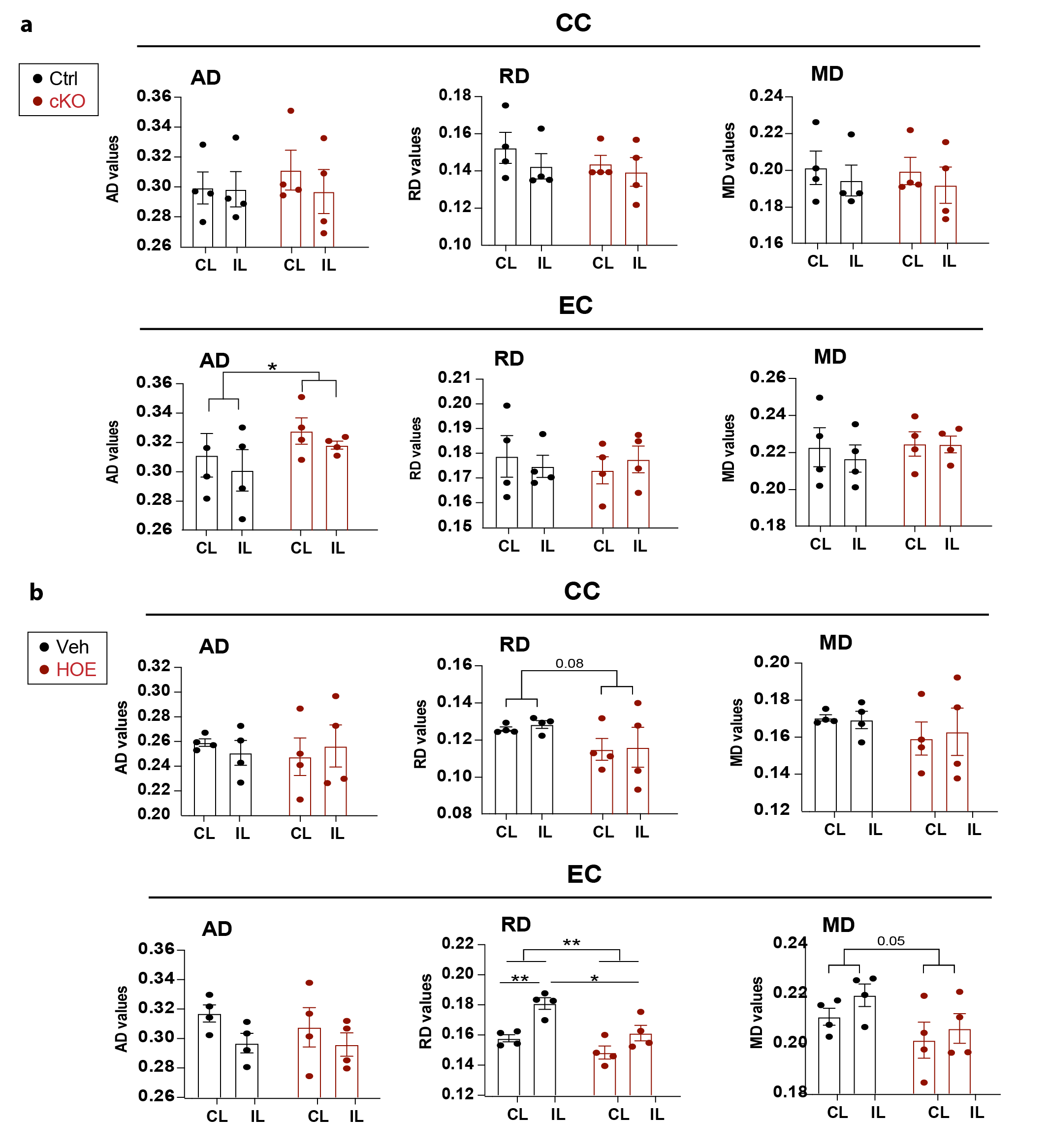
**

**Fig. S10. Additional parameters of DTI in microglial *Nhe1* cKO and HOE642-treated mice at 30 days post-TBI.**

**a.** Analysis of axial diffusivity (AD), radial diffusivity (RD) and medium diffusivity (MD) in the white matter tracts (CC and EC) of the same cohort of mice in Fig. 7a-d. **b**. Analysis of AD, RD and MD in CC and EC of the same cohort of mice in Fig. 7e-h. Data are mean ± SEM. N = 4. * p < 0.05, ** p < 0.01.


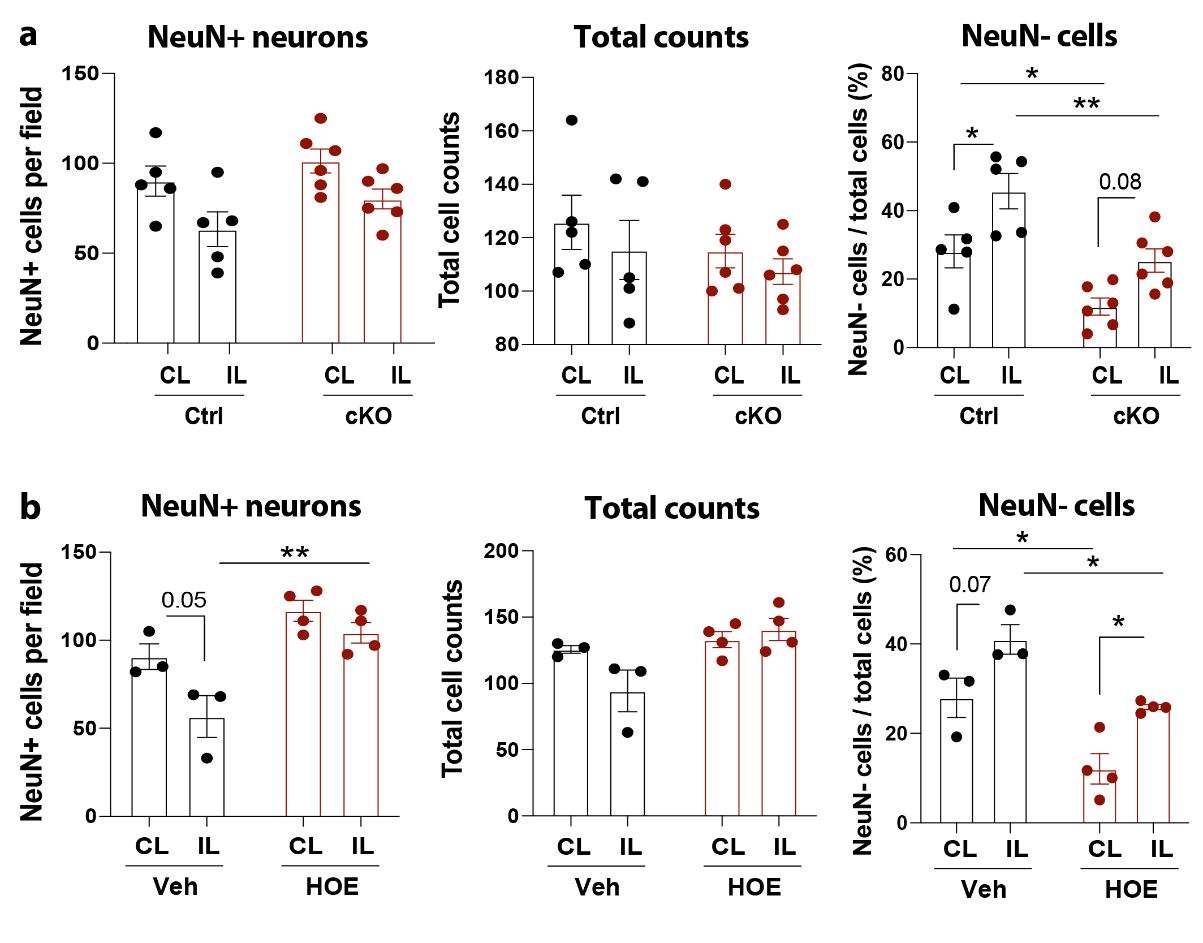


**Fig. S11. Additional analysis of perilesion neurons in microglial *Nhe1* cKO or HOE642-treated mice at 3 days post-TBI.**

**a.** Absolute counts of NeuN^+^ neurons and DAPI^+^ total cells, as well as NeuN^-^ cells percentage from the same cohort of data as in Fig. 1d. **b.** Absolute counts of NeuN^+^ neurons and DAPI^+^ total cells, as well as NeuN^-^ cells percentage from the same cohort of data as in Fig. 6b. * p < 0.05, ** p < 0.01.


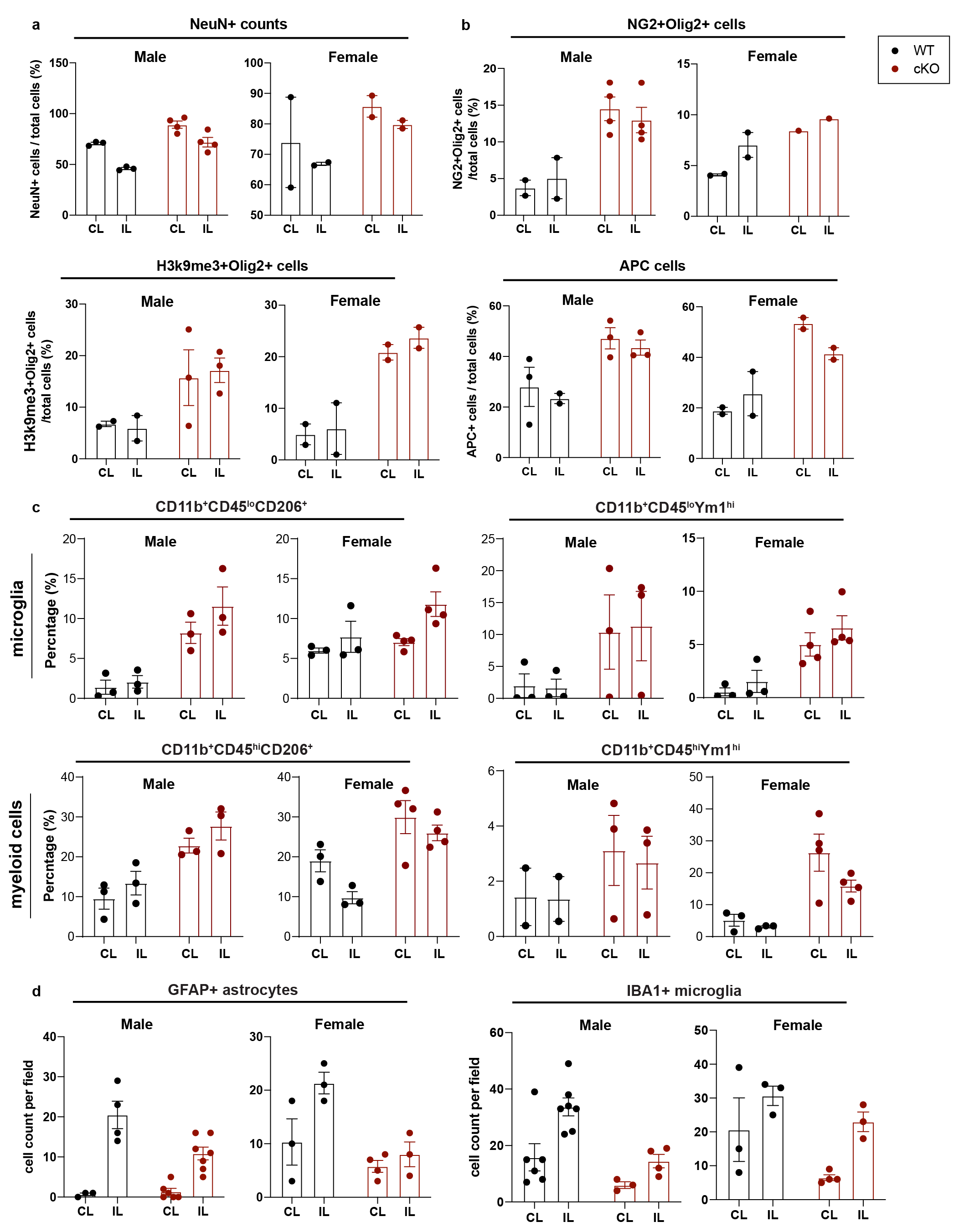


**Fig. S12. Sex-dependent differences in Ctrl or cKO mice at 3 days post-TBI.**

Similar trends of changes were observed between males and females in Ctrl or cKO mice at 3 days post-TBI. **a.** Sex-dependent differences in the same cohort of data as in Fig. 1d. **b.** Sex-dependent differences in the same cohort of data as in Fig. 2b-c. **c.** Sex-dependent differences in the same cohort of data as in Fig. 3c-d. **d.** Sex-dependent differences in the same cohort of data as in Fig. 3e.

**Table S1. Primer sequences used in RT-qPCR**

| **Gene** | **Forward** | **Reverse** |
| --- | --- | --- |
| *S1pr1* | TTGAGCGAGGCTGCTGTTTC | GGGGTGGTATTTCTCCAGGC |
| *Psen2* | GAAGACTCCTACGACAGTTTTGG | CACCAGGACGCTGTAGAAGAT |
| *Ifi206* | GGCATCCTGATTCCTCCACCA | CTGGCAGACACGGTTCAGAAG |
| *Igsf8* | CTGCTAATACTTGGAACCAGGTG | ACGTTGCAGGAGATAGAGACA |
| *Ppia* | TGTGCCAGGGTGGTGACTTT | CGTTTGTGTTTGGTCCAGCAT |

**Table S2. List of antibodies used in immunostaining and flow** **cytometry**

| **Antibody** | **Host** | **Dilution** | **Company** | **Catalog No.** | **Application** | |
| --- | --- | --- | --- | --- | --- | --- |
| MAP2 | Mouse | 1:200 | EMD Millipore | MAB3418 | Immuno-staining | |
| NeuN | Rabbit | 1:200 | Abcam | ab177487 |  |  |
| MBP | Rabbit | 1:200 | Abcam | ab40390 |  |  |
| NG2 | Rabbit | 1:200 | EMD Millipore | AB5320 |  |  |
| Ki67 | Rabbit | 1:500 | EMD Millipore | ab15580 |  |  |
| Caspase3 | Rabbit | 1:200 | Cell Signaling Technology | 9662S |  |  |
| H3K9me3 | Rabbit | 1:200 | Abcam | ab8898 |  |  |
| Olig2 | Mouse | 1:200 | EMD Millipore | MABN50 |  |  |
| APC | Mouse | 1:200 | EMD Millipore | OP80 |  |  |
| GFAP | Mouse | 1:200 | Cell Signaling Technology | 3670S |  |  |
| IBA1 | Rabbit | 1:200 | Wako | 019-19741 |  |  |
| BUV395-CD11b | Rat | 250 | BD Biosciences | 563553 | Flow Cytometry |  |
| BV510-CD45 | Rat | 250 | BioLegend | 103138 |  |  |
| eFluor 450-CD16/32 | Rat | 250 | Invitrogen | 48-0161-82 |  |  |
| Alexa Fluor 700-CD86 | Rat | 250 | BD Biosciences | 560581 |  |  |
| PE-Cy7-CD206 | Rat | 250 | Invitrogen | 25-2061-82 |  |  |
| PE-Ym-1 | Rabbit | 2500 | Abcam | ab211621 |  |  |

**References**

1. Percie du Sert N, Hurst V, Ahluwalia A, Alam S, Avey MT, Baker M, et al. The ARRIVE guidelines 2.0: Updated guidelines for reporting animal research. J Cereb Blood Flow Metab. 2020;40(9):1769-77.

2. Song S, Wang S, Pigott VM, Jiang T, Foley LM, Mishra A, et al. Selective role of Na(+) /H(+) exchanger in Cx3cr1(+) microglial activation, white matter demyelination, and post-stroke function recovery. Glia. 2018;66(11):2279-98.

3. Hashimoto D, Chow A, Noizat C, Teo P, Beasley MB, Leboeuf M, et al. Tissue-resident macrophages self-maintain locally throughout adult life with minimal contribution from circulating monocytes. Immunity. 2013;38(4):792-804.

4. Valny M, Honsa P, Kirdajova D, Kamenik Z, Anderova M. Tamoxifen in the Mouse Brain: Implications for Fate-Mapping Studies Using the Tamoxifen-Inducible Cre-loxP System. Front Cell Neurosci. 2016;10:243.

5. Fogg DK, Sibon C, Miled C, Jung S, Aucouturier P, Littman DR, et al. A clonogenic bone marrow progenitor specific for macrophages and dendritic cells. Science. 2006;311(5757):83-7.

6. Ajami B, Bennett JL, Krieger C, Tetzlaff W, Rossi FM. Local self-renewal can sustain CNS microglia maintenance and function throughout adult life. Nat Neurosci. 2007;10(12):1538-43.

7. Parkhurst CN, Yang G, Ninan I, Savas JN, Yates JR, 3rd, Lafaille JJ, et al. Microglia promote learning-dependent synapse formation through brain-derived neurotrophic factor. Cell. 2013;155(7):1596-609.

8. Begum G, Song S, Wang S, Zhao H, Bhuiyan MIH, Li E, et al. Selective knockout of astrocytic Na(+) /H(+) exchanger isoform 1 reduces astrogliosis, BBB damage, infarction, and improves neurological function after ischemic stroke. Glia. 2018;66(1):126-44.

9. Lee E, Hwang I, Park S, Hong S, Hwang B, Cho Y, et al. MPTP-driven NLRP3 inflammasome activation in microglia plays a central role in dopaminergic neurodegeneration. Cell Death Differ. 2019;26(2):213-28.

10. Zhang B, Zou J, Han L, Beeler B, Friedman JL, Griffin E, et al. The specificity and role of microglia in epileptogenesis in mouse models of tuberous sclerosis complex. Epilepsia. 2018;59(9):1796-806.

11. Schafer DP, Heller CT, Gunner G, Heller M, Gordon C, Hammond T, et al. Microglia contribute to circuit defects in Mecp2 null mice independent of microglia-specific loss of Mecp2 expression. Elife. 2016;5.

12. Theroux P, Chaitman BR, Erhardt L, Jessel A, Meinertz T, Nickel WU, et al. Design of a trial evaluating myocardial cell protection with cariporide, an inhibitor of the transmembrane sodium-hydrogen exchanger: the Guard During Ischemia Against Necrosis (GUARDIAN) trial. Curr Control Trials Cardiovasc Med. 2000;1(1):59-67.

13. Sen T, Gupta R, Kaiser H, Sen N. Activation of PERK Elicits Memory Impairment through Inactivation of CREB and Downregulation of PSD95 After Traumatic Brain Injury. J Neurosci. 2017;37(24):5900-11.

14. Bouet V, Boulouard M, Toutain J, Divoux D, Bernaudin M, Schumann-Bard P, et al. The adhesive removal test: a sensitive method to assess sensorimotor deficits in mice. Nat Protoc. 2009;4(10):1560-4.

15. Shelton SB, Pettigrew DB, Hermann AD, Zhou W, Sullivan PM, Crutcher KA, et al. A simple, efficient tool for assessment of mice after unilateral cortex injury. J Neurosci Methods. 2008;168(2):431-42.

16. Darwish H, Hasan H. Y-Shaped Maze to Test Spontaneous Object Recognition and Temporal Order Memory After Traumatic Brain Injury. Methods Mol Biol. 2019;2011:383-92.

17. Zhao H, Nepomuceno R, Gao X, Foley LM, Wang S, Begum G, et al. Deletion of the WNK3-SPAK kinase complex in mice improves radiographic and clinical outcomes in malignant cerebral edema after ischemic stroke. J Cereb Blood Flow Metab. 2017;37(2):550-63.

18. Song S, Yu L, Hasan MN, Paruchuri SS, Mullett SJ, Sullivan MLG, et al. Elevated microglial oxidative phosphorylation and phagocytosis stimulate post-stroke brain remodeling and cognitive function recovery in mice. Commun Biol. 2022;5(1):35.
